# High‐Performing Clean Water Production by Rational Design of Functional Solar Evaporator and Vapor Condensation

**DOI:** 10.1002/advs.202505008

**Published:** 2025-05-20

**Authors:** Bingxing Wu, Xiangqian Fan, Chaorui Xue, Qing Chang, Jinlong Yang, Shengliang Hu, Haolan Xu

**Affiliations:** ^1^ Research Group of New Energy Materials and Devices State Key Laboratory of Coal and CBM Co‐Mining North University of China Taiyuan 030051 P. R. China; ^2^ State Key Laboratory of New Ceramics and Fine Processing Tsinghua University Beijing 100084 P. R. China; ^3^ Future Industries Institute UniSA STEM University of South Australia Adelaide South Australia 1067 Australia

**Keywords:** carbon dot, interfacial solar evaporation, invert‐structured vapor condenser, MXene, photothermal conversion, reduced graphene oxide, water purification

## Abstract

Interfacial solar evaporation is a promising technology to address the global issue of water scarcity with a minimum carbon footprint. Although solar‐to‐vapor conversion efficiency has been significantly improved, it does not actually lead to high clean water production due to low vapor condensation efficiency and water collection rate, which hinders real‐world applications. Herein, an invert‐structured solar evaporation and vapor condensation device coupled with solar evaporators featuring special vertically aligned vapor diffusion channels is designed to target both high evaporation and water collection rates. Graphene oxides, carbon dots, and MXene are used to construct sophisticated nanostructure to effectively confine the thermal energy in the structure for water evaporation. The vertical channels in the evaporators allow downward vapor transportation for condensation. The bottom condenser, made of highly thermal‐conductive materials with hydrophobic coating, is cooled by bulk water underneath, accelerating the dropwise condensation processes. In addition, since vapor is pushed downward, light absorption on the top evaporation surface is not declined. Both top and bottom evaporation surfaces are activated for water evaporation. Therefore, this inverted device achieves a record‐high water‐collection rate of 2.31 kg m^−2^ h^−1^ under one sun, superior to conventional single‐stage solar evaporation systems, suggesting great potential in practical seawater desalination.

## Graphical abstract

An invert‐structured vapor condensation device coupled with solar evaporators featuring special vertically aligned vapor diffusion channels is developed to target both high evaporation and water collection rates. By minimizing the optical loss caused by condensed water and speeding up the dropwise condensation process, it achieves a record‐high water‐collection rate, far superior to a conventional single‐stage solar water purifier.

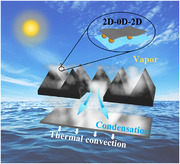

## 1 Introduction

Water scarcity is regard as a major issue affecting human survival and global economic development.^[^ ^1^, ^2^ ^]^ Solar‐driven interfacial water evaporation (SDIE) has attracted much attention as a sustainable technology for seawater desalination, wastewater purification, and water–electricity cogeneration with a minimized carbon footprint, benefiting access to clean water.^[^ ^3^, ^4^, ^5^, ^6^ ^]^ The past decade has witnessed significant improvement in solar evaporation performance through the rational design of functional photothermal materials and evaporators.^[^ ^7^, ^8^ ^]^ Although many materials with broadband light absorption, such as graphene‐based materials and MXene, can maximize solar‐to‐heat conversion efficiency, it is inconceivable that their evaporation rates cannot reach as high as expected.^[^ ^9^, ^10^, ^11^, ^12^ ^]^ The reason is only a part of the solar‐converted heat contributed to the phase transition of water from liquid to vapor occurring at liquid–air interfaces.^[^ ^13^, ^14^ ^]^ To trigger vaporization, a minimum energy is needed to allow the water molecules to escape from the surface of the bulk water into air. If this threshold is not reached by heating, the thermal energy is easily consumed by liquid water and the environment without effective water evaporation.^[^ ^15^, ^16^, ^17^ ^]^ Therefore, to improve solar‐converted heat utilization efficiency for water evaporation, the structure of photothermal materials should be designed to trap and accumulate the heat inside of the materials instead of dissipating into the environment during photothermal conversion processes.^[^ ^18^, ^19^ ^]^

Typical 2D photothermal materials (e.g., graphene, MXene) usually confront the issue of high emissivity in the infrared range, which often leads to a thermal radiation loss, thereby reducing the overall photothermal efficiency.^[^ ^20^, ^21^ ^]^ To suppress their infrared emission, the interband transition of electrons should be efficiently blocked by introducing 0D nanoparticles/clusters anchored on their surface.^[^ ^22^, ^23^ ^]^ Eco‐friendly carbon dots (CDs), as a typical 0D carbon nanomaterial, possess many advantages including low cost, excellent chemical stability, and tunable light absorption and conversion.^[^ ^24^ ^]^ In addition, CDs can combine with a variety of nanomaterials through their surface functional groups. Therefore, rationally architecting sophisticated structures (e.g., 2D‐0D‐2D) using 0D CDs and 2D photothermal materials will greatly improve light absorption, photothermal conversion, heat confinement and utilization, benefiting superior solar water evaporation.^[^ ^8^, ^25^, ^26^ ^]^ Furthermore, similar to many reported photothermal materials^[^ ^15^, ^18^, ^27^ ^]^ CDs with rich hydrophilic groups also have the ability to reduce the water evaporation enthalpy by regulating water state,^[^ ^28^, ^29^ ^]^ thus further improving solar evaporation rates.

Apart from high evaporation rates, the efficiency of vapor condensation is another crucial factor that determines clean water production.^[^ ^30^, ^31^, ^32^, ^33^ ^]^ In many reported solar evaporation and water collection devices, vapor condensation generally takes place on the surfaces of a transparent cover located above the evaporator.^[^ ^33^, ^34^, ^35^ ^]^ In such enclosed systems, condensation of vapor occurs while the temperature falls below the dew point. If vapor condensation is inefficient, the humidity level inside the device becomes high which impedes continuous water evaporation, consequently diminishing both evaporation rate and water collection rate. However, once the vapor is condensed on the transparent cover, the formed water droplets not only obstruct light absorption, resulting in a high optical loss up to 35%, but also serve as thermal barriers, further curtailing the vapor condensation rate. These factors collectively cause the reduced water collection rate and clean water production, with solar‐to‐collected water conversion efficiency of only ∼35%.^[^ ^36^, ^37^, ^38^, ^39^, ^40^ ^]^ To address these challenges, it is virtually important to develop a strategy to regulate the vapor diffusion path and condensation zone via rational design of coupled functional evaporators and solar evaporation‐condensation devices.

In this work, we fabricated a novel functional evaporator (named as GCM@MS) by assembling reduced graphene oxides (rGO), CDs and MXene on the skeleton of commercial melamine sponge (MS). Benefiting from the photothermal components and the formed 2D‐0D‐2D structure, the GCM@MS was endowed with the reduced vaporization enthalpy, excellent heat confinement and highly efficient heat transfer from photothermal materials to adjacent water, resulting in more rapid solar water evaporation. Thanks to excellent mechanical properties and processability of the GCM@MS, top‐down vapor diffusion channels were made to couple the invert‐structured evaporation‐vapor condensation device to significantly improve water collection rates. Because vapor is condensed at the bottom of the device, optical loss at the top is significantly reduced. Moreover, the bottom vapor condenser made by highly thermal‐conductive materials with hydrophobic coating can be cooled by bulk water underneath and facilitate latent heat dissipation, speeding up the dropwise condensation process. Therefore, this inverted condensation device achieves a water‐collection rate of 2.31 kg m^−2^ h^−1^, which is 4.6‐7.7 times higher than that of a conventional single‐stage solar water purifier. This substantially improved water collection rate directly increases clean water production, paving the way for practical applications.

## 2 Results and Discussion

**Figure 1:**
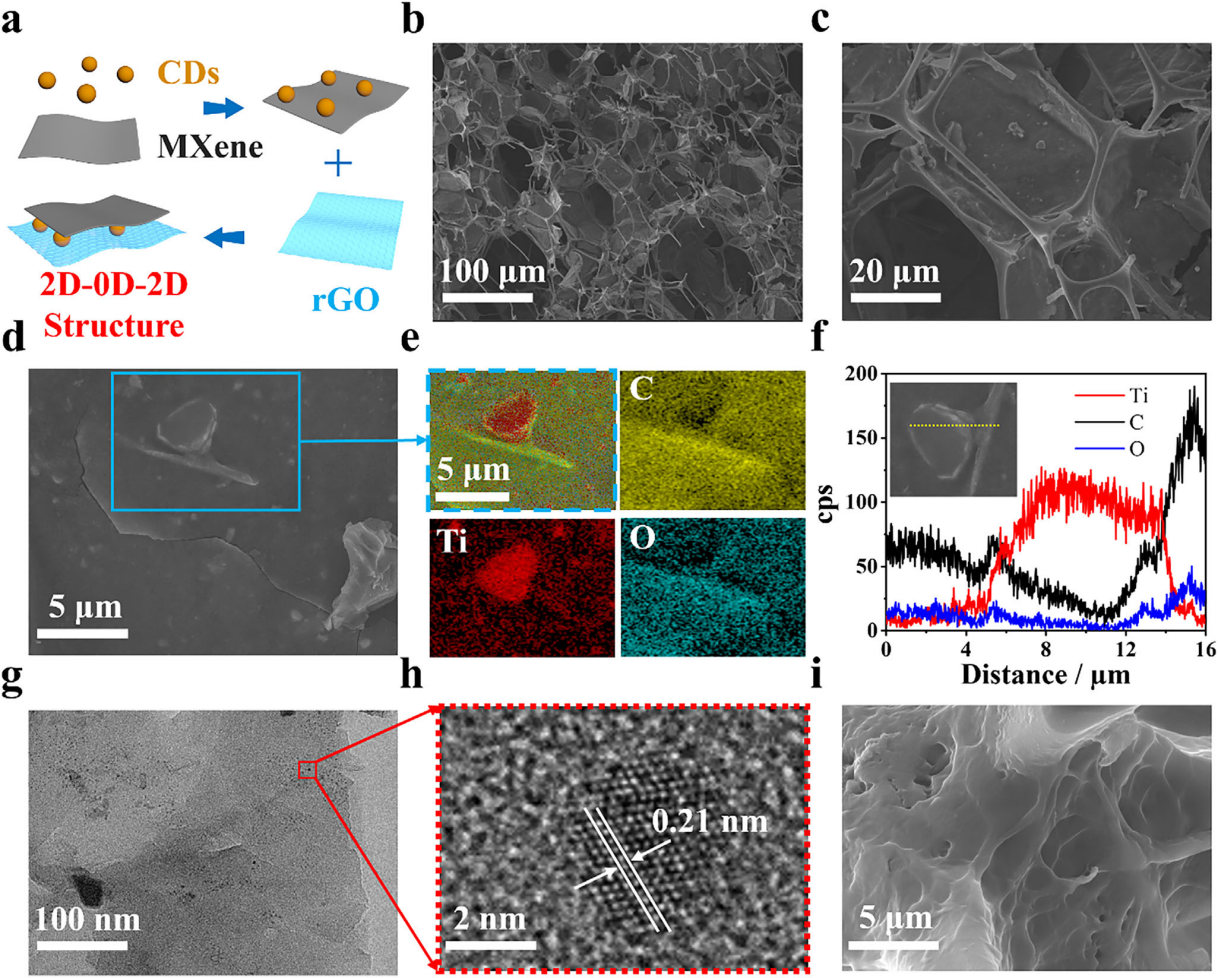
Materials preparation and characterization. a) Schematic of GCM preparation using MXene, CDs and rGO; b–e) SEM images of GCM with different magnifications and the corresponding EDS mapping; f) Elemental line‐scan profile shown in the inset; g) TEM image of GCM; h) HRTEM image of a typical carbon nanoparticle in GCM; i) SEM image of H‐GCM@MS.

The evaporator was fabricated by successively coating rGO, and CDs‐MXene on the skeletons of the melamine sponge (MS) followed by PVA hydrogel stabilization (**Figure** **1**a; Figure S1, Supporting Information). Scanning electron microscopy (SEM) characterization showed that the pristine MS had an open porous framework with pore sizes of 100–200 µm (Figure S2a–c, Supporting Information). After coating of rGO (rGO@MS), the original open porous structure of MS retained while many wrinkle stripes were observed on the surfaces of the skeletons (Figure S2d–f, Supporting Information). The energy‐dispersive spectroscopy (EDS) mapping of rGO@MS depicted the even distribution of both C and O elements across the skeletons of MS (Figure S3a–d, Supporting Information), indicating that the MS was fully covered by rGO nanosheets. The well‐exfoliated MXene nanosheets (Figure S4, Supporting Information) were obtained by etching the MAX‐Ti_3_AlC_2_ with HCl/LiF.^[^ ^23^ ^]^ Then CDs were adsorbed on the surface of MXene nanosheets to produce 2D‐0D structures (i.e., CDs‐MXene). By immersing the rGO@MS into the CDs‐MXene suspension, a 2D‐0D‐2D photothermal layer composed of rGO, CDs, and MXene was formed on the MS surface (GCM@MS). The X‐ray diffraction (XRD) pattern of GCM@MS (Figure S5, Supporting Information) presented all the characteristic peaks of MXene with a clear left shift of (002) plane compared to that of MAX‐Ti_3_AlC_2_,^[^ ^41^ ^]^ confirming the presence of MXene nanosheets in the GCM. SEM image and EDS element mapping showed micron flakes and Ti element originated from MXene (Figure 1b‐e). EDS line‐scan analysis (Figure 1f) clearly depicted the distribution of MXene and CDs, with Ti element specifically presented on the micron flake while C element distributed throughout the entire scan scope. Furthermore, transmission electron microscopy (TEM) confirmed that CDs were well dispersed on the surfaces of MXene sheets (Figure 1g). The observed lattice space of 0.21 nm could assign to the (100) facets of graphite (Figure 1h). Therefore a 2D‐0D‐2D photothermal structure was formed. By in situ polymerization of polyvinyl alcohol (PVA) in the channels of GCM@MS, the skeletons introduced numerous micron grooves with widths ranging from 1–10 µm (Figures 1i; S2g–i, Supporting Information), verifying that the coatings (i.e., rGO, MXene, and CDs) on the skeletons of MS were effectively fixed by the interpenetrated PVA hydrogels (H‐GCM@MS).

**Figure 2:**
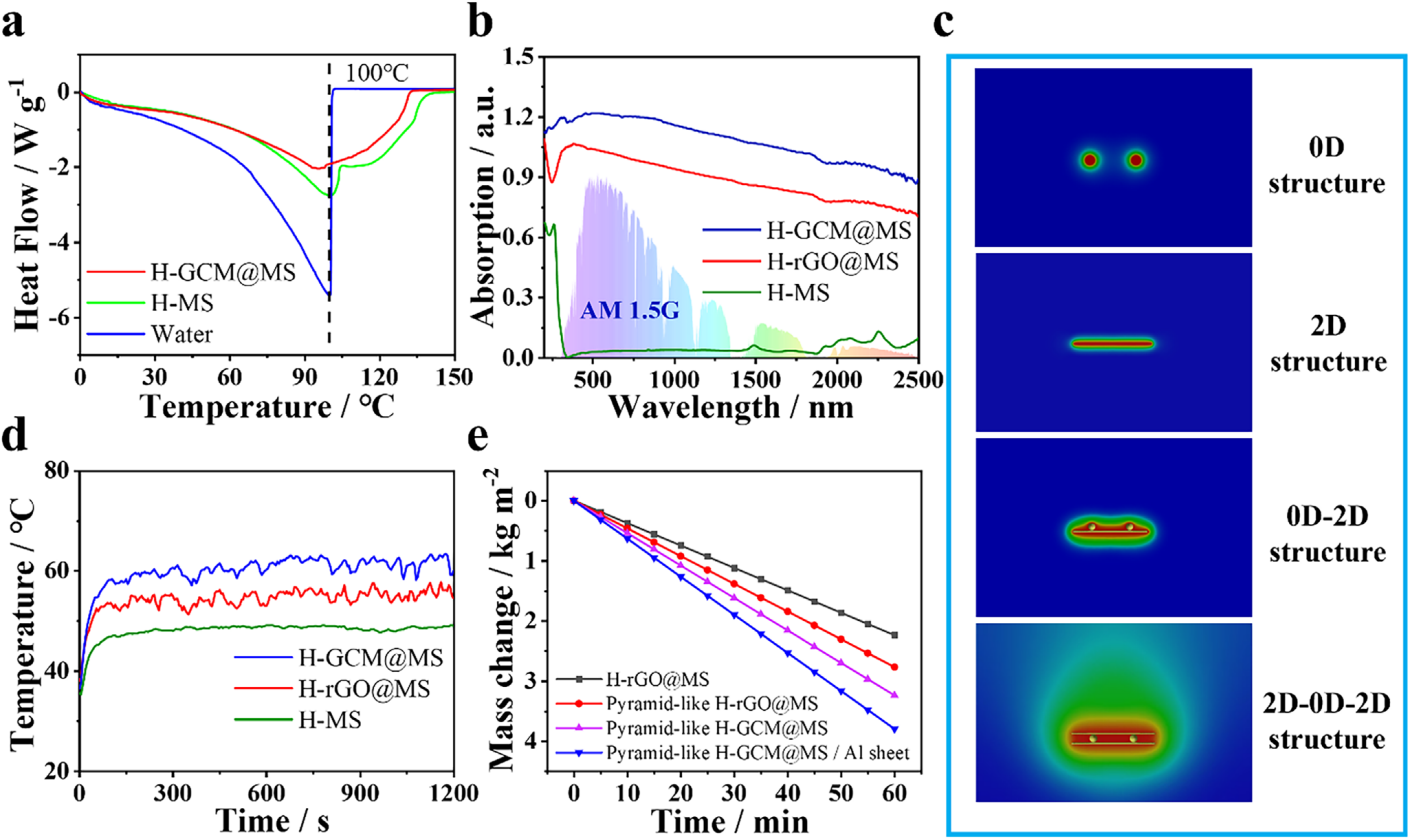
Optical and photothermal conversion properties. a) DSC curves of pure water, H@MS and H‐GCM@MS; b) UV–vis‐NIR absorption spectra of H‐MS, H‐rGO@MS and H‐GCM@MS; c) Simulated thermal confinement effects of different material structures; d) Surface temperatures of H‐MS, H‐rGO@MS and H‐GCM@MS as a function of time under one sun irradiation; e) Mass changes of water versus time for the evaporators with different shapes under one sun irradiation.

The differential scanning calorimetry (DSC) was further used to determine the vaporization enthalpy of water in the evaporators (**Figure** **2**a). The H‐GCM@MS showed the lowest vaporization enthalpy of 1129 J g^−1^, which was merely 45% that of bulk water (2503 J g^−1^). Moreover, the equivalent vaporization enthalpy of the water in H‐GCM@MS was measured by evaporating water under dark condition. The obtained equivalent vaporization enthalpy of the water in H‐GCM@MS by experimental method was very close to the DSC measured results (Figure S6, Supporting Information). According to the UV–visible‐near infrared (UV–Vis‐NIR) spectra (Figure 2b), the H‐GCM@MS exhibited excellent light absorption across the range of the entire solar spectrum, superior to that of hydrogel stabilized rGO@MS (H‐rGO@MS) and MS (H‐MS). Apart from efficient solar light capture, promoting vapor production using the ambient solar flux (*γ*_solar_) requires remarkable reduction of the heat losses from the receiver. The evaporation rate *ṁ* can be described by,

(1) $$\begin{array}{ccc} \dot{m}\;h_{\mathrm{fg}} & = & \beta \gamma_{\mathrm{solar}}A-Q_{\mathrm{loss}}=h_{\mathrm{eva}}\;A\;\left(T_{\mathrm{interf}}-T_{\mathrm{amb}}\right)\\ & = & \varphi h_{m}A\left(T_{m}-T_{\mathrm{water}}\right) \end{array}$$

where *ṁ*, *h*_fg_, *β*, *γ*_solar_, *A, Q*_loss_, *h*_eva,_ *T*_interf,_ and *T*_amb_ represent the evaporation rate, latent heat, light absorption of the evaporator, the radiation intensity of the light, the surface area of the evaporator, total heat loss, equivalent heat transfer coefficient at the water‐air interface, water‐air interface temperature, and ambient temperature, respectively. *φ*, *h*_m_, *T*_m_ and *T*_water_ are the ratio of the heat consumed by liquid water phase change and the heat transferred to liquid water, the equivalent heat transfer coefficient at the interface of the photothermal materials and liquid water, the local temperature at the surface of photothermal materials and the temperature of liquid water, respectively. Assuming an identical solar input, the evaporation rate is proportional to the surface temperature of photothermal materials according to the Equation (1). Figure 2c depicted simulated thermal gradient contours of different photothermal structures, which revealed that the solar‐converted heat was effectively confined and accumulated in the layers of 2D‐0D‐2D structure. In comparison, single 0D, 2D, and 0D‐2D structures exhibited relatively weak heat confinement effects due to direct and fast thermal dissipation into the environment. Therefore, the H‐GCM@MS could acquire a higher surface temperature *T*_m_ to enhance the heat transfer efficiency to water nearby to boost water evaporation under solar irradiation.

Under one sun in the air, the surfaces of H‐GCM@MS, H‐rGO@MS, and H‐MS were rapidly heated to an equilibrium temperature within 100 s (Figure 2d; Figure S7, Supporting Information), where the equilibrium temperature of H‐GCM@MS was the highest (≈63 °C). To further improve the light energy harvesting, evaporators with different surface topographies were designed (Figure S8, Supporting Information). It was found that under one sun, the evaporators with sharp pyramid‐like topography delivered water evaporation rates up to 30% higher than that of flat evaporators (Figure 2e; Figure S8a,b, Supporting Information). The highest evaporation rate of the pyramid‐like H‐GCM@MS reached 3.29 kg m^−2^ h^−1^ (Figure 2e), significantly higher than that of pyramid‐like evaporators prepared using single component (H‐rGO@MS 2.74 kg m^−2^; H‐CDs@MS 1.63 kg m^−2^ h^−1^; H‐MXene@MS 2.64 kg m^−2^ h^−1^) and bicomponent (H‐(rGO‐MXene)@MS 3.04 kg m^−2^ h^−1^; H‐(rGO‐CDs)@MS 2.90 kg m^−2^ h^−1^; H‐(MXene‐CDs)@MS 2.78 kg m^−2^ h^−1^) (Figure S9 and Table S1, Supporting Information). Additionally, the evaporation rate was influenced by CDs contents in H‐GCM@MS (Figure S10, Supporting Information). All of these control experimental results verified the advantage of the 2D‐0D‐2D structures in photothermal conversion, energy confinement and water evaporation. To adapt the evaporator to the invert‐structured solar evaporation‐ condensation device, a number of millimeter‐sized holes were drilled (Figure S8c, Supporting Information) on the pyramid‐like H‐GCM@MS to allow downward vapor diffusion. To take advantage of the solar light pass through the holes, a reflective aluminum plate was placed underneath (Figure S8c,d, Supporting Information). An even higher evaporation rate of 3.79 kg m^−2^ h^−1^ was achieved (Figure 2c; Figure S11, Supporting Information) corresponding to a solar‐to‐vapor energy conversion efficiency of 97.11% (Figure S6, Supporting Information), which exceeded most of the reported evaporators (Figure S12 and Table S2, Supporting Information).^[^ ^9^, ^42^, ^43^, ^44^, ^45^, ^46^, ^47^, ^48^, ^49^, ^50^ ^]^ In addition, our evaporator showed excellent self‐cleaning function for continuous desalination and water purification (Figure S13, Supporting Information). The evaporation rate kept unchanged in brines with salinity ranging from 0 to 21.0% during 7 days of continuous operation. In addition, the evaporator also presented an excellent function of organic pollutants degradation during solar evaporation of dye‐polluted water.

**Figure 3:**
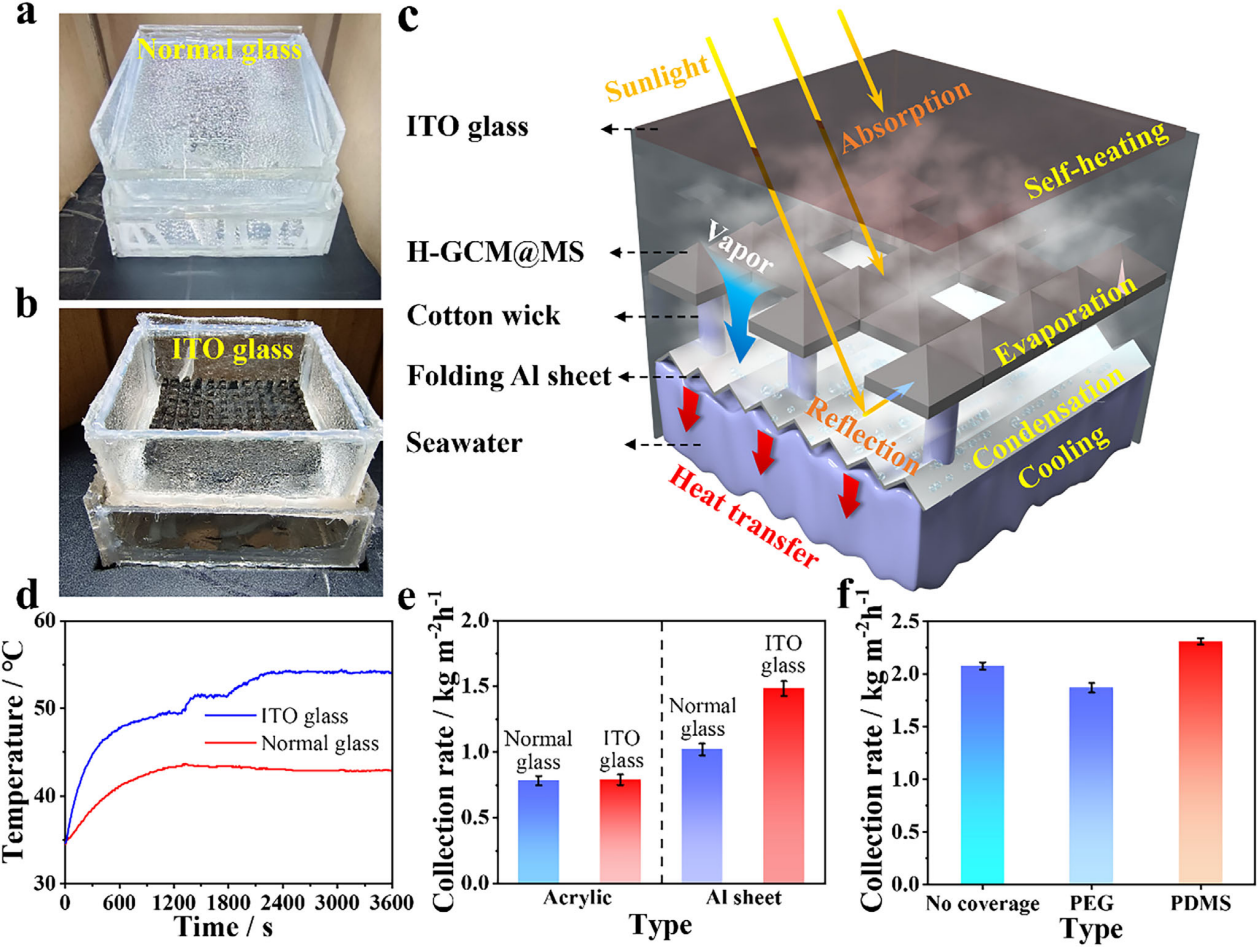
Condensation collection device. a and b) Photographs of conventional collection device and invert‐structured vapor condensation device during water evaporation; c) Schematic illustration of the structure and working principle of the invert‐structured vapor condensation device; d) Surface temperature curves of the top cover of ITO glass and ordinary glass under light irradiation; e) Effects of the types of condenser and top cover on water collection rates; f) Effects of surface coatings of the Al sheet on water collection rates.

There is no doubt that the evaporation rate is not equivalent to the clean water collection rate since the generated vapor has to undergo a condensation process for water collection.^[^ ^37^, ^40^, ^51^ ^]^ The conventional solar evaporation and condensation devices, which are generally constructed using an enclosed chamber with a transparent roof for both sunlight passage and vapor condensation, often show low vapor condensation efficiency and compromised light harvest.^[^ ^33^, ^34^, ^52^ ^]^ As illustrated in **Figure** **3**a, numerous condensed water droplets were quickly formed on the transparent roof of the device during solar evaporation and water collection. These droplets significantly obstructed the sunlight absorption of the evaporators underneath. Meanwhile, the latent heat of vapor condensation was also released in the chamber and increased the temperature in the chamber. Consequently, both evaporation and condensation efficiencies, as well as the water collection rate, were diminished notably. In order to eliminate such adverse effects, we designed an inverted solar evaporation‐condensation device which took advantages of the channels in the evaporators for downward vapor transportation and condensation (Figure 3b,c). The vapor was efficiently condensed on the surface of a thermal conductive aluminum (Al) sheet at the bottom, with one side in direct contact with bulk water to maintain a lower surface temperature relative to the vapor temperature. More importantly, to avoid any vapor condensation and droplet formation on the top transparent roof, indium tin oxide (ITO) glass which can absorb part of UV and NIR light to heat itself was utilized (Figure S15, Supporting Information). As shown in Figure 3d, under one sun illumination, the equilibrium temperature of the ITO glass cover reached ≈55 °C, which was ≈12 °C higher than that of the normal glass cover. The ITO surface temperature was also very close to the temperatures of the evaporators and the generated vapor (Figure S16, Supporting Information), thus effectively hindering vapor condensation on the top roof (Figure 3b). As a result, the light harvest of the evaporators did not decline. It should be pointed out that compared to the reported inverted design, where one side of the 2D evaporator is generally attached to the top transparent roof and only the bottom evaporation surface contributes to water evaporation, in our inverted device, the evaporator is suspended in the middle, and all the evaporation surfaces implement water evaporation (Figure 3c). The generated vapor was pushed downward through the channels in the evaporators for condensation on the Al condenser underneath (Figure 3c). This new inverted single‐stage condenser delivered a water collection rate of 1.48 kg m^−2^ h^−1^ (Figure 3e), which was nearly twice that of the conventional condensation devices.^[^ ^53^, ^54^, ^55^ ^]^ In comparison, devices with normal glass cover and lower thermally conductive acrylic condenser delivered much lower water collection rates (Figure 3e). The water collection rate could be further improved to 1.89 kg m^−2^ h^−1^ via simply folding the Al sheet to increase the condensation surface area and heat dissipation area (Figure S17, Supporting Information). The temperature of the bulk water also influenced heat dissipation and vapor condensation efficiency. A lower bulk water temperature is conducive to the dissipation of released latent heat from vapor condensation, resulting in a higher water collection rate (Figure S18, Supporting Information).

The vapor condensation efficiency could be further enhanced via surface modification of the Al surface. Since the hydrophobicity of a surface could affect water contact angel and thermal resistance for vapor condensation, the wettability of the condensation surface was adjusted by coating hydrophilic polyethylene glycol (PEG) and hydrophobic polydimethylsiloxane (PDMS). The contact angle of PDMS‐modified Al sheet was 103.14°, much higher than that of PEG‐modified Al sheet (33.02°) (Figure S19a–c, Supporting Information). Apparently, vapor was prone to condense on the hydrophobic surface (Figure S19d–f, Supporting Information). As a result, the hydrophobic PDMS‐modified Al sheet endowed an enhanced water collection rate (2.31 kg m^−2^ h^−1^) relative to those of the pristine and PEG‐modified Al sheets (Figure 3f) and is a record high among the reported single‐stage devices.

**Figure 4:**
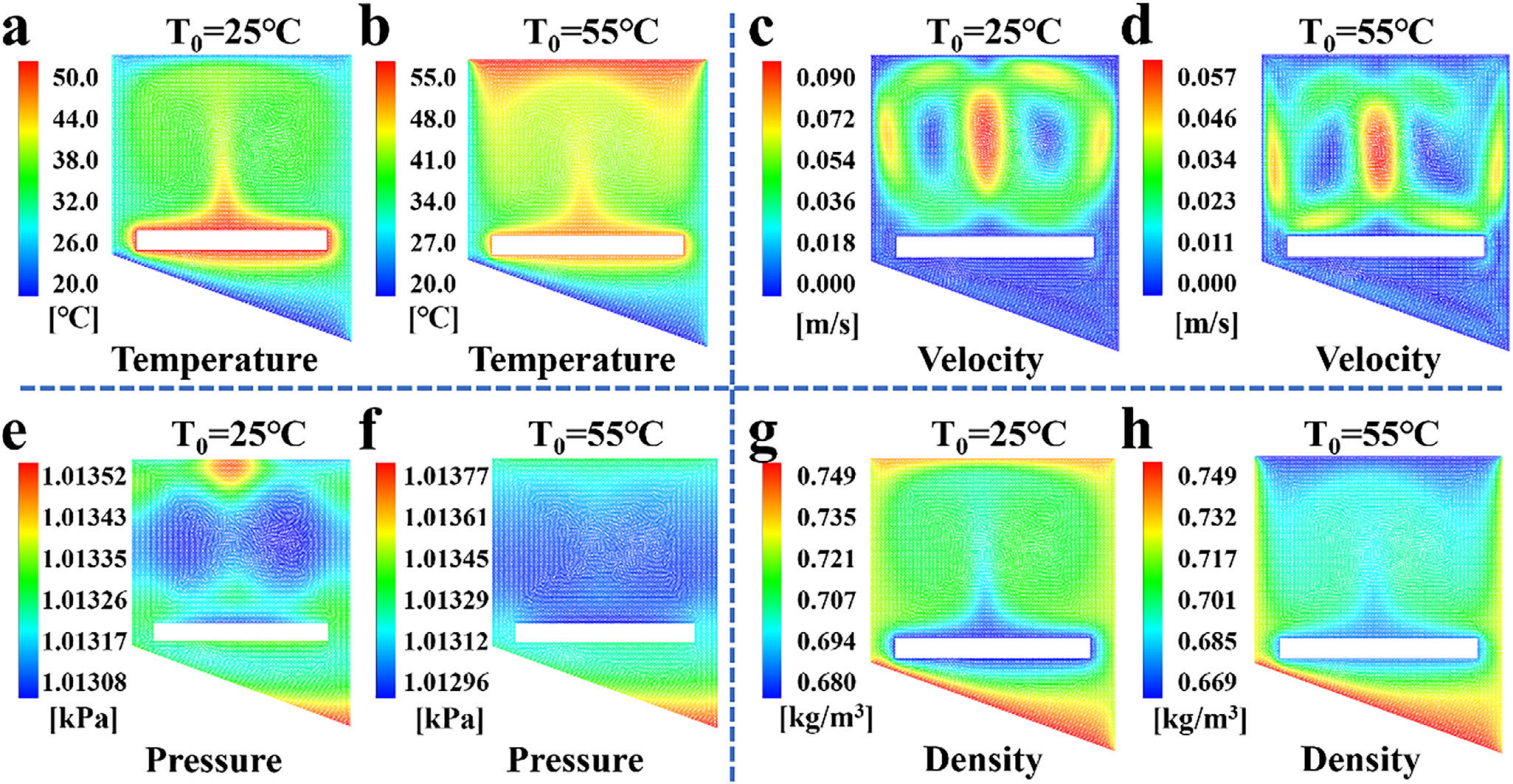
Theoretical simulation analysis. a and b) The temperature distributions inside water collection device at the initial temperature of 25 and 55 °C for top cover; c and d) The heat flow velocity distributions inside water collection device at the initial temperature of 25 and 55 °C for top cover; e and f) The vapor pressure distributions inside water collection device at the initial temperature of 25 and 55 °C for top cover; g and h) The vapor density distributions inside water collection device at the initial temperature of 25 and 55 °C for top cover.

Numerical simulations were conducted to investigate the temperature distribution, heat flow, vapor pressure, and distribution in the inverted solar evaporation‐condensation device, since these factors determine the vapor condensation efficiency and water collection rate. Two controlled cases were investigated with the top cover temperatures of the device set as 25 and 55 °C, respectively. The simulated temperature distribution patterns indicated that both cases had a distinct low‐temperature region under the evaporators (**Figure** **4**a,b). This region was a relative stagnation zone in the condensation chamber according to the vapor velocity distribution contour map (Figure 4c,d). A symmetrical vapor flow/convection emerged in velocity distribution contour map for the low temperature of top surface, while it was not formed in the case of the high top surface temperature. This implied that more vapor accessed the low‐temperature region at the bottom when the top cover surface was with a high temperature, promoting vapor condensation. The distribution contour maps of the vapor pressure and density (Figure 4e–h) offered a more intuitive view of the migration of vapor from the top zone to the bottom, realized by simply adjusting the temperature of the top surface of the condensation chamber. With a high top surface temperature, it can be observed that the space above the evaporator sustained more even pressure and lower vapor density, indicating the low regional humidity, which could benefit high evaporation rates of the evaporators. Thereby, all the simulated results supported the facts of high evaporation rate of the evaporators and high vapor condensation efficiency of the bottom Al sheet in our inverted single‐stage solar evaporation‐condensation device.

**Figure 5:**
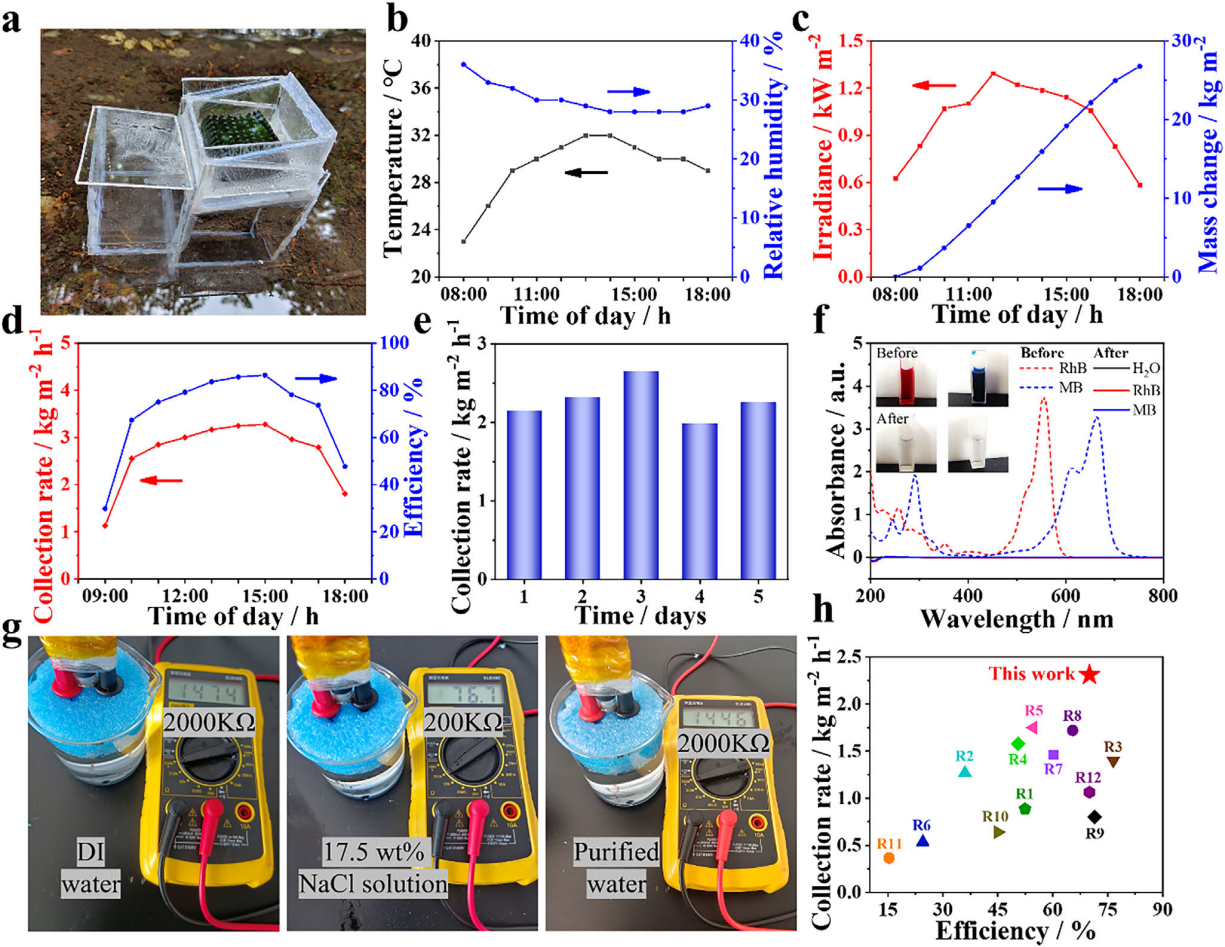
Performance evaluation of the condensation collection device. a) Photograph of the collection device; b) Outdoor temperature and relative humidity recorded over time on a sunny day from 08:00 to 18:00; c) The solar radiation over time and the corresponding water evaporation performance of H‐GCM@MS; d) Water collection rate and efficiency of the collection device; e) Water collection rates measured during five consecutive days; f) The capability of H‐GCM@MS to remove organic dyes of RhB and MB; g) Determination of collected water purity by the multimeter with a constant distance between electrodes; h) Performance comparison of our collection device with the reported results.

To assess its potential for practical applications, the performance of the inverted single‐stage device was tested under outdoor conditions (Figure S20, Supporting Information). An evaporator with a diameter of 10 cm and a thickness of 1.2 cm was used to evaporate brine and dye‐contaminated water in the device (**Figure** **5**a). The tests were performed from 08:00 to 18:00 (September 3, 2024, at the North University of China) under natural sunlight, and the environmental temperature, humidity as well as solar irradiation intensity were monitored and recorded (Figure 5b,c; S21, Supporting Information). A total water collection of 26.8 L m^−2^ was achieved (Figure 5c). The water collection rates, and collection efficiencies were calculated at different times (Figure 5d), where the highest values (water collection rate of 3.27 kg m^−2^ h^−1^ and collection efficiency of 86.5%) occurred at ≈3:00 pm. To confirm the stability and durability, the device was operated during daytime for consecutive 5 days. The device continuously produced clean water every day, while the average clean water production rate fluctuated due to the change in weather conditions. For the dye‐contaminated water, after solar evaporation purification, no residual dyes were detected in the collected clean water (Figure 5f). For desalination, the resistance value of collected water from brine evaporation was similar to that of the commercial purified water in the market (Figure 5g and S22, Supporting Information). These results confirm that our device is applicable for the treatment of various water source to produce high quality clean water. More importantly, our device delivers record‐high water collection rate, superior to the reported single‐stage solar evaporation systems (Figure 5h; Table S3, Supporting Information)^[^ ^32^, ^34^, ^42^, ^49^, ^50^, ^53^, ^54^, ^55^, ^56^, ^57^, ^58^, ^59^ ^]^

## 3 Conclusion

This work achieves both high solar evaporation and water collection rates via the rational design of the structure of photothermal materials and evaporators as well as the inverted evaporation‐condensation device. By assembling rGO, CDs and MXene to form 2D‐0D‐2D structured photothermal materials, a higher internal temperature was realized under solar light irradiation which improved the efficiency of heat transfer from the photothermal materials to adjacent liquid water for rapid water evaporation. The photothermal evaporators were modified with vertical channels for downward vapor diffusion to couple with the invert‐structured vapor condensation device for efficient vapor condensation at the bottom. In addition, the top transparent cover was specifically designed with a relatively higher surface temperature to push downward migration of vapor and avoid water droplet formation on its surface. The suspended configuration of the evaporators ensured that all the surfaces contributed to water evaporation. The bottom condensation surface was directly contacted with bulk water for cooling and was modified with hydrophobic PDMS to boost dropwise condensation. Collectively, these advantages resulted in a record‐high water‐collection rate of 2.31 kgm^−2^ h^−1^ among the single‐stage solar water purifiers. Outdoor tests confirm the excellent performance of this device for real‐world applications for seawater desalination and waste water purification.

## 4 Experimental Section

Preparation of the evaporator (Figure S1, Supporting Information): Typically, sponges were first processed into the desired shape and size, followed by soaking in the suspension of GO with a concentration of 10 g L^−1^ for 2 h, and subsequent dying at 60 °C in an oven. After that, the GO coated sponges reacted with HI solution (≥45.0 wt%) at 95 °C for 40 s. The obtained samples were washed three times with deionized water and absolute ethanol, respectively, followed by drying at 60 °C for another 6 h to obtain the rGO‐coated sponges (rGO@MS). Next, the resultant samples were soaked into a mixed solution containing MXenes (2 g L^−1^) and CDs (20 g L^−1^) for 1 h and then dried at 80 °C for 6 h. Thereafter, 5 mL of the aqueous solution of polyvinyl alcohol (PVA, 0.65 g) and glutaraldehyde (125 µL, 50% wt. in DI water) was brushed on the surface of the samples, followed by spraying HCl solution (1.2 M) to conduct gelation. The obtained samples were washed two times with deionized water, followed by freezing in a refrigerator and then thawed at room temperature. Finally, hydrogel‐stabilized coatings of rGO, CDs, and MXene on sponges were achieved to serve as the evaporators (H‐GCM@MS).

### Statistical Analysis

The data processing was conducted using Origin Pro software (OriginLab Corp.). The data about water evaporation results, clean water collection rates, and DSC results are presented as the average and standard deviation of three tests per sample.

## Conflict of Interest

The authors declare no conflict of interest.

## Supporting information



Supporting Information

## Data Availability

The data that support the findings of this study are available from the corresponding author upon reasonable request.
