# Supplementary material for: High‐Performing Clean Water Production by Rational Design of Functional Solar Evaporator and Vapor Condensation
Source: Adv Sci (Weinh). 2025 May 20;12(30):e05008. doi: 10.1002/advs.202505008 (PMC12376612; doi:10.1002/advs.202505008)
Supplement: Supplementary file 1 — Supporting Information [file ADVS-12-e05008-s001.docx]

((Supporting Information can be included here using this template))

Supporting Information

High-Performing Clean Water Production by Rational Design of Functional Solar Evaporator and Vapor Condensation

*Bingxing Wu, Xiangqian Fan, Chaorui Xue, Qing Chang, Jinlong Yang, Shengliang Hu* and Haolan Xu**

**1. Experimental details**

**1.1 Materials and Chemicals**

Commercial porous melamine sponge (MS) was purchased from Xijie Home Furnishings Company of China without further treatment. Aluminum sheet was purchased from Dongguang Yongcheng Technology Co., Ltd. Chemical reagents including HI, formic acid (88 v/v%), concentrated sulfuric acid (98 wt%), concentrated phosphoric acid (85 wt%), concentrated hydrochloric acid (12 M), glutaraldehyde (GA, 50 wt% in the water) and anhydrous ethanol (AEA) were purchased from Damao Chemical Reagent Factory, China. MAX-Ti_3_AlC_2_ was purchased from Jilin 11 Technology Co., Ltd. Graphite powders, Polydimethylsiloxane (PDMS), Polyethylene glycol (PEG, average Mn 6000), Methyl blue (MB), Rhodamine B (RhB), Lithium fluoride (LiF), hydrogen peroxide (H_2_O_2_, 30 v/v%) and polyvinyl alcohol (PVA, Mw ~75,000) were purchased from Shanghai Aladdin Biochemical Technology Co., Ltd., China. All chemical reagents were directly used without further purification. The indium tin oxide (ITO) glass was purchased from Suzhou Shangyang Solar Energy Technology Co., Ltd.

**1.2 Preparation of materials**

The preparation of graphene oxides (GO) followed the modified Hummer method.^[S1]^ Carbon dots (CDs) were obtained by a selective oxidation etching of coal tar pitch as reported in our previous work. ^[S2]^ The suspension of MXenes (Ti_3_C_2_T_x_) was prepared by the previously reported method. ^[S3]^ Typically, 2 g of lithium fluoride was mixed with 40 mL of hydrochloric acid (9 M) in a polytetrafluoroethylene beaker with a volume of 100 mL under stirring. Then 2 g of MAX-Ti_3_AlC_2_ was slowly added into the beaker (Note: the reaction is intense). The reaction proceeded for 24 hours at 35 ℃ under stirring. After centrifugation, the obtained precipitate was washed for several times till the pH of the supernatant liquid reached 5. The precipitate was dispersed in 40 ml of ethanol by ultrasonication. Finally, the black brown upper liquid containing Ti_3_C_2_T_x_ nanosheets was collected by the centrifugal separation (10000 rpm, 10 min).

**1.3 Sample characterization**

The surface morphology of the sample was imaged using a scanning electron microscope (ZEISS Sigma 300, Germany). The crystal structure of the materials were examined by a transmission electron microscope (TEM, JEOL JEM-F200, Japan) operated at 20 kV. Absorption spectra were measured using a UV-Vis-NIR spectrophotometer (Hitachi UH4150, Japan) with an integrated sphere in the wavelength range of 250-2500 nm. X-ray diffraction (XRD) patterns were recorded on an X-ray diffractometer (DX-2700BH). The evaporation enthalpy of the sample was measured by differential scanning calorimetry (HENVEN HSC-, China). The contact angle measurement was conducted by Kunshan Shengding SDC 350KS. A multimeter (VICTOR201, VICTOR Instrument) was used to test the resistance of the water samples. The surface temperature of the solar evaporator was detected by a dual-channel thermometer (Xinster HT-9815) and an infrared camera (Fotric 323+, Shanghai).

**1.4 Solar water evaporation experiment**

The evaporators were placed into home-made thermal insulation polystyrene foam floating on water in a cylinder container filled with water or brine. An appropriate hole was drilled in the foam to host cotton threads for water transportation. The container was placed on an electronic balance with an accuracy of 0.0001 g for real-time monitoring of the variation of the solution mass during solar evaporation, and the resultant data were recorded to determine the evaporation rates based on the projection area. A solar simulator (CEL-HXUV300, CEAULIGHT, China) with an AM1.5 G optical filter was employed as the light source and the power density was confirmed by a power meter (China Education Au-light, CEL-NP2000, China).

The solar-to-vapor conversion efficiency (𝜂) was calculated by the following equation as reported: ^[S4]^

$\eta=\frac{\dot{m}H_{e}}{C_{\mathrm{opt}}q_{0}}$ (S1)

where $\dot{m}$, C_opt_, and $q_{0}$represent the evaporation rate, optical concentration and solar irradiation intensity, respectively. *H*_e_ is the evaporation enthalpy of the water in the evaporator, which was measured and calculated by the following equation: ^[S5]^

$H_{e}m_{e}=H_{0}m_{0}$ (S2)

in which *H*_0_ and *m*_0_ refer to the evaporation enthalpy and the evaporation rate of bulk water, and *m*_e_ is the evaporation rate of the evaporator in dark conditions, respectively.

**1.5 Water collection setup**

The solar evaporation-condensation device consisted of a top cover, solar evaporators and a bottom condenser. The top cover was made by indium tin oxide (ITO) glass (1 mm thickness) and placed above the solar evaporators. The distance from the top surface of the evaporator to the ITO glass was set as 40 mm. The bottom condenser located 10 mm under the solar evaporators and the tilt angle of the condensation slope was set as 15°. The condenser was made of aluminum plate. The outer size of the device was 140 mm in length, 85 mm in width and 140 mm in height. Five outlets were designed for water collection.

**2. Modeling of theoretical simulation**

2.1 Heat manganement of photothermal materials

The heat transfer in different structures of photothermal materials could be described by the equation given below:

$E_{i}=\int_{V_{s}} {\rho_{s}C}_{s}\left( T_{s}-T_{0} \right)ⅆV+\int_{0}^{t} \int_{A_{i}} q_{w}ⅆAⅆt$ (S3)

$E_{i}=\beta\gamma_{\mathrm{solar}}A$ (S4)

where *E_i_* represents the overall heat input; *ρ_s_*, *C*_s_ and *T_s_* are the density, thermal capacity and temperature, respectively; *V*_s_ is the volume of the structure; *T*_0_ is the initial temperature; *A*_i_ is the contact area between the material and the liquid water; *q*_w_ is the convective heat transfer density, which can be represented by the heat flux: $q_{w}=h(T_{s}-T_{w})$, where *h* is the equivalent heat transfer coefficient, and *T*_w_ is the temperature of the bulk water;  *β* is the absorption ratio of the solar absorber; $\gamma_{\mathrm{solar}}$ is the radiation intensity of the light; *A* is the light absorption area of the material.

We ignored the impact of thermal radiation in numerical calculations. The incident solar energy intensity was set to 100 mW cm^-2^. The initial temperature field for the entire region was set to an ambient temperature of 293 K. The velocity field is the initial velocity of 0 m s^-1^ in the fluid domain.

2.2 Heat transfer process of invert-structured vapor condenser

Considering solar-converted heat as the total energy input for the whole system and a stable evaporation rate at the surface of evaporator, the heat transfer is described by the following equation,

$E_{i}=\rho_{v}C_{v}\frac{\partial T_{v}}{\partial t}+\rho_{v}C_{v}v_{v}\cdot\nabla T_{v}+\nabla\cdot\left( \alpha_{v}\nabla T_{v} \right)+\left( \frac{\partial P_{v}}{\partial t}+v_{v}\cdot\nabla P_{v} \right)+\nabla\cdot\left( \alpha_{g}\nabla T_{g} \right)$ (S5)

where *E_i_* represents the overall heat input; *ρ*_v_, *C*_v_ and *T*_v_ are the density, thermal capacity and the local temperature of vapor, respectively; $\nabla$ represents the divergence operator; *P*_v_ is the vapor pressure; *ν*_v_ is the velocity; *α*_v_ and *α*_g_ are thermal conductivity of the condensation wall and the top glass, respectively; *T*_g_ is the temperature of the top glass. The flow field of vapor in the system is described by mass conservation and momentum conservation equations for time-dependent incompressible gas,

$\frac{\partial\rho_{v}}{\partial t}+\nabla\cdot(\rho_{v}v_{v})=0$ (S6)

$\rho_{v}\left( \frac{\partial v_{v}}{\partial t}+v_{v}\cdot\nabla v_{v} \right)=-\nabla P_{v}+\nabla\cdot\left[ v_{v}\left( \nabla v_{v}+\nabla{v_{v}}^{T_{v}} \right) \right]+\rho_{v}g$ (S7)

The convection of vapor in the system is originated from the changes of density gradient and pressure with temperature *T*_v_, which can be expressed as:

$\frac{dP_{v}}{dT_{v}}=\frac{H_{e}}{T_{v}\Delta V}$ (S8)

$\rho_{v}=\frac{P_{v}}{RT_{v}}$ (S9)

where *H_e_* is the enthalpy of phase transition; ∆*V* is the specific volume change during the phase transition process, *R* and *g* are the gas constant and the gravitational acceleration, respectively.

We assumed that the solar-converted thermal energy was transferred to the condensation walls via evaporation-condensation process and inner convective heat transfer. Meanwhile, the heat at the condensation walls was transferred to bulk water mainly through thermal conduction, which can be expressed by a heat flux: *q*(*T_v_*) = *h* (*T_v_* –*T*_out_), where *h* is the equivalent heat transfer coefficient between the condenser and bulk water, *T*_out_ is the outer temperature of condenser walls. Note that the thermal radiation energy of the whole system could be neglected. The model was also numerically calculated under steady and transient analysis mode. To perform a qualitative analysis, both the temperature of surroundings and the initial temperature of the side walls were set as room temperature 25 °C. The initial temperature of bulk water at the bottom of the aluminum plate was given as 20 °C, while the temperature of of the top cover was set as 25 °C and 55°C for ordinary glass and ITO glass, respectively. The incident solar energy intensity was set as 100 mW cm^-2^. The simulation domain was resolved by 30490 elements with tetrahedra as small as 0.52 mm. A refined mesh was applied to the boundary of condenser walls and macrochannels. In the transient mode simulation, the initial and final conditions of transient analysis were t = 1 s and t =3600 s, respectively.

**3. Supporting results**


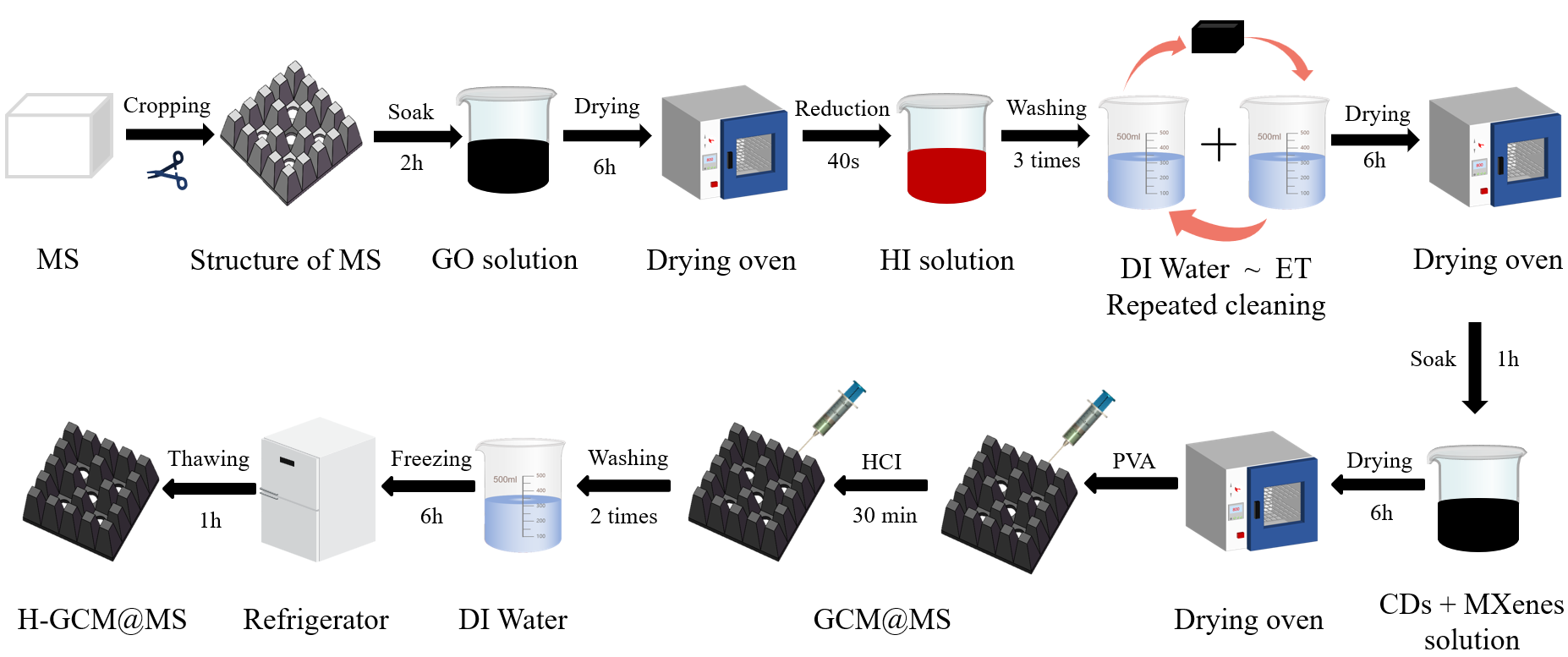


**Figure S1**. Schematic illustration of H-GCM@MS preparation processes.

**
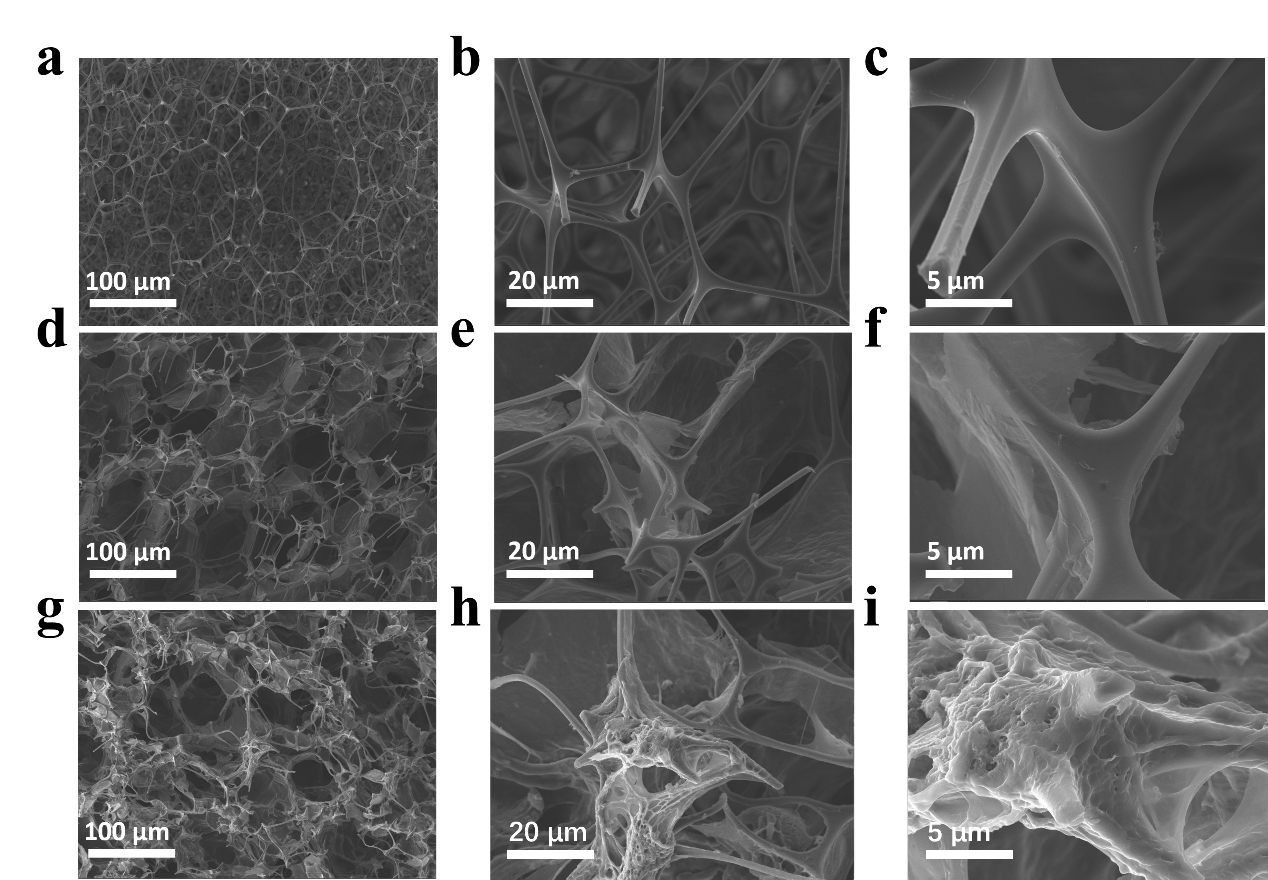
**

**Figure S2.** SEM images of (a-c) MS; (d-f) rGO@MS; (g-i) H-GCM@MS with different magnifications

**
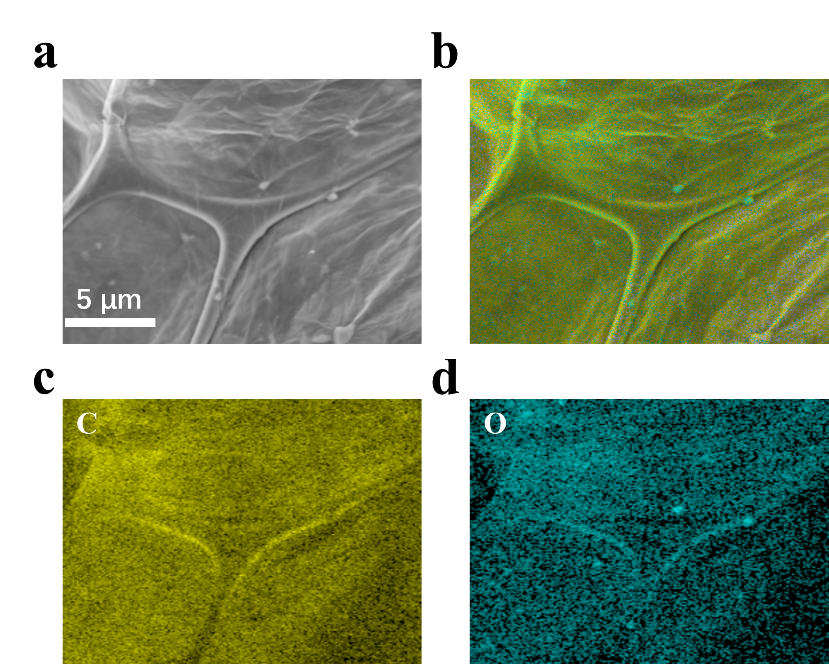
**

**Figure S3.** SEM image of the rGO@MS and the corresponding EDS mapping.


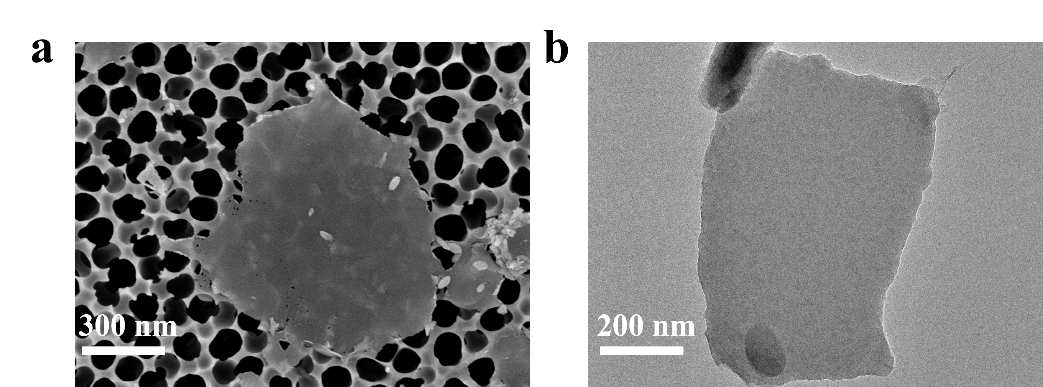


**Figure S4.** (a) SEM and (b) TEM images of the as-prepared MXene nanosheets.

**
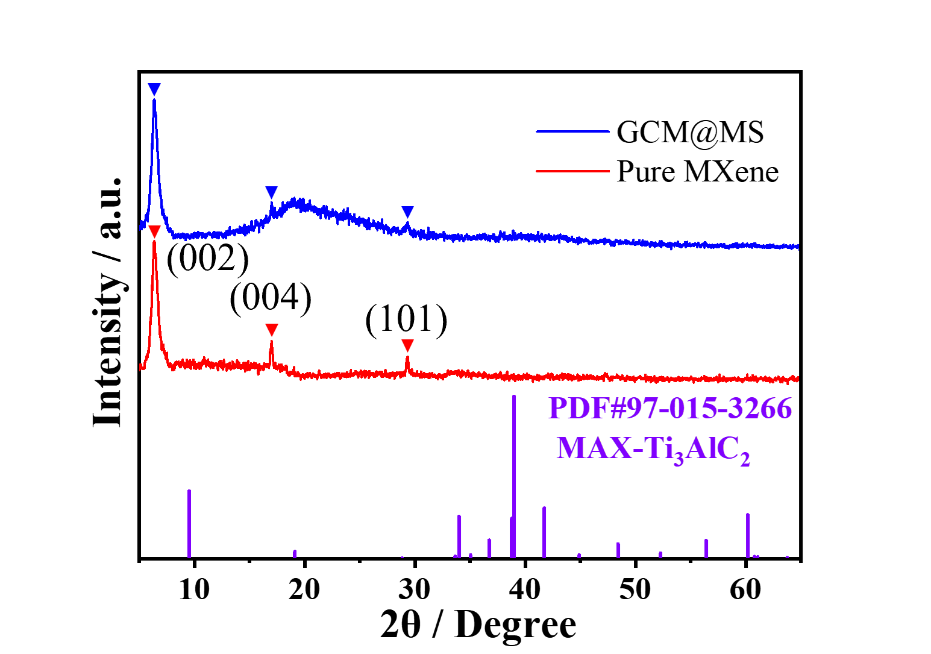
**

**Figure S5.** XRD patterns of the as-prepared MXene nanosheets and GCM@MS

**
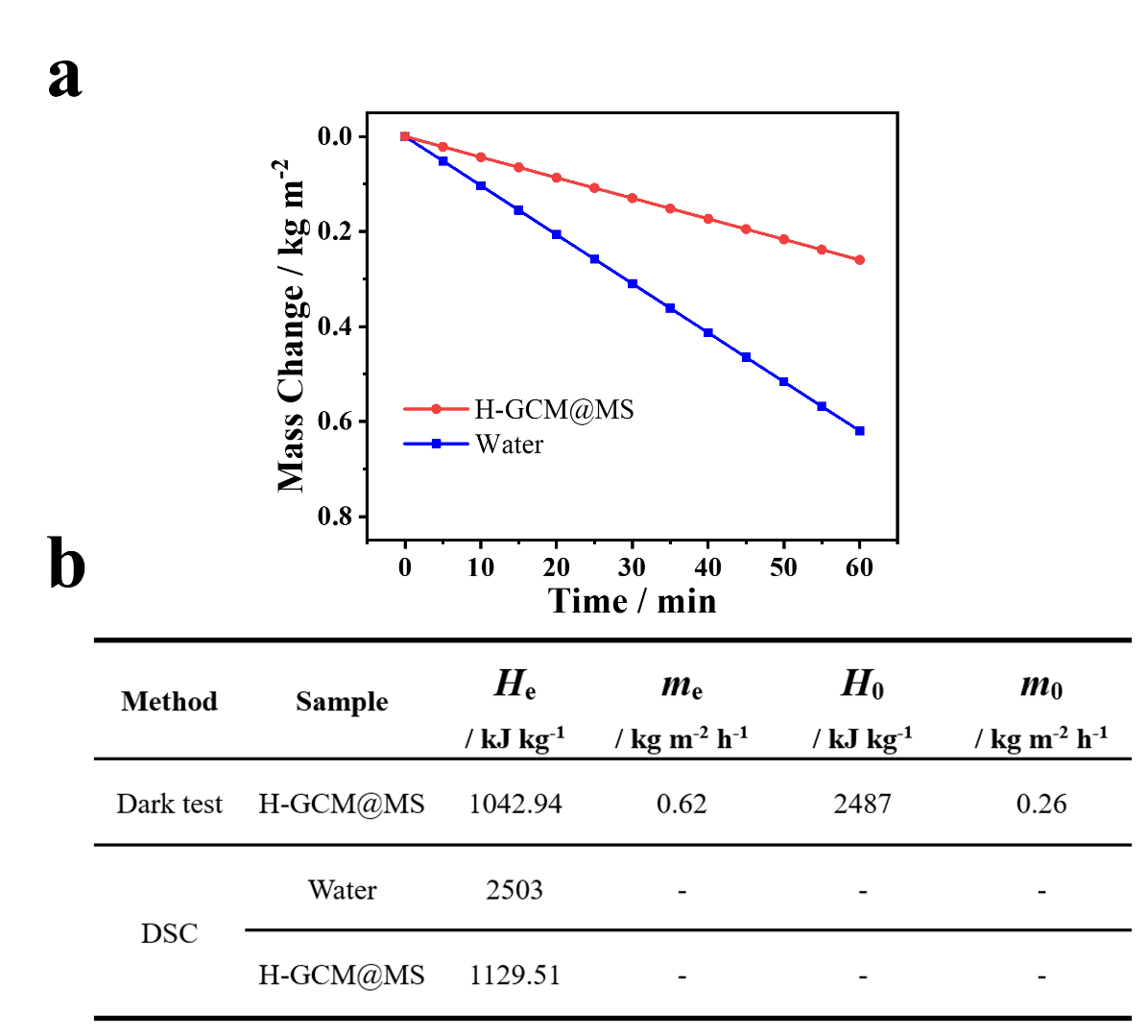
**

**Figure S6.** (a) Evaporation rates of bulk water and water in H-GCM@MS tested under dark condition; (b) Experimental results tested by DSC and dark evaporation.

Note: According to the equation (S1) and the obtained evaporation enthalpy (*H*_e_), the solar-to-steam energy conversion efficiency of H-GCM@MS is 97.11%.

**
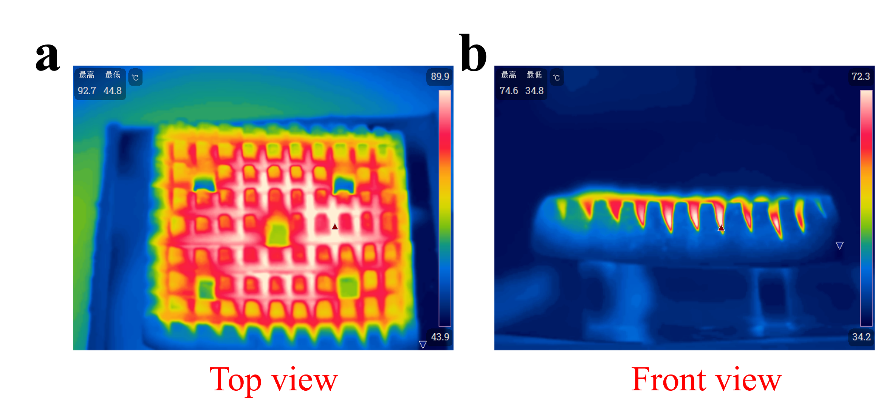
**

**Figure S7.** IR images of H-GCM@MS under solar irradiation

**
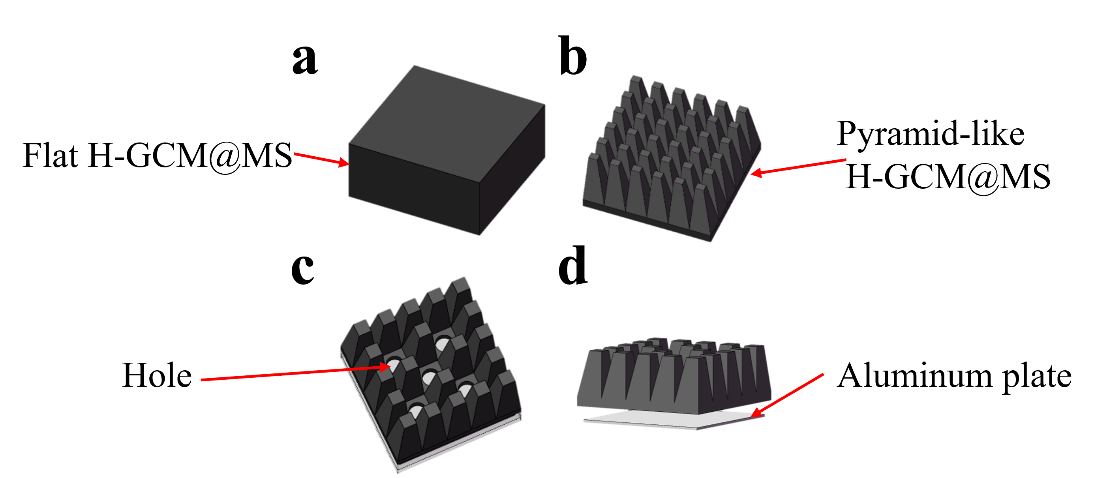
**

**Figure S8.** Schematic illustration of the solar evaporators with different structures


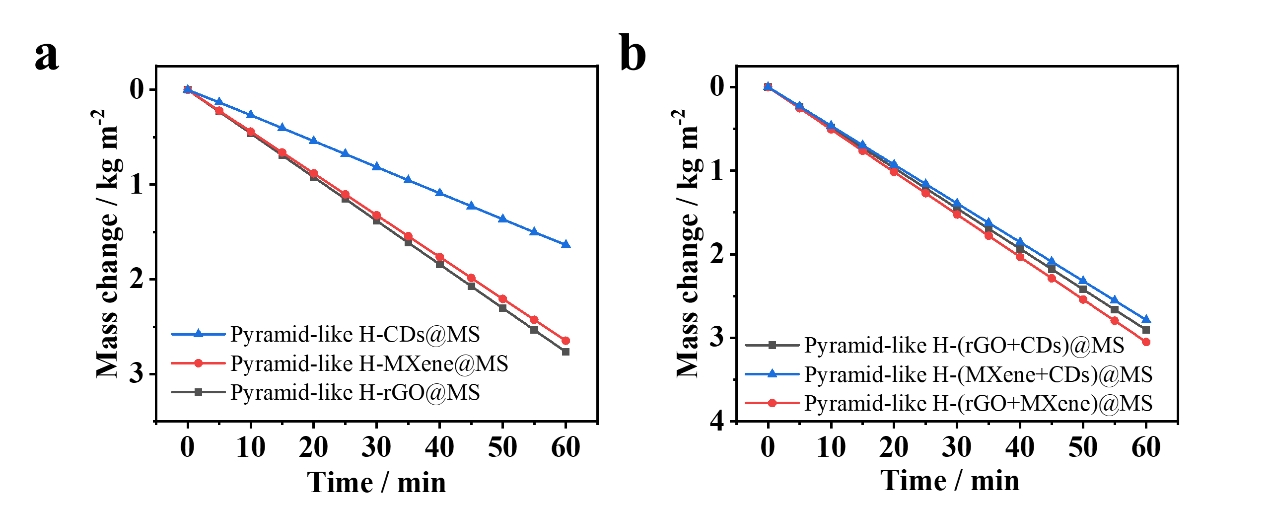


**Figure S9.** Evaporation performances for (a) three single-component structures and (b) three two-component structures


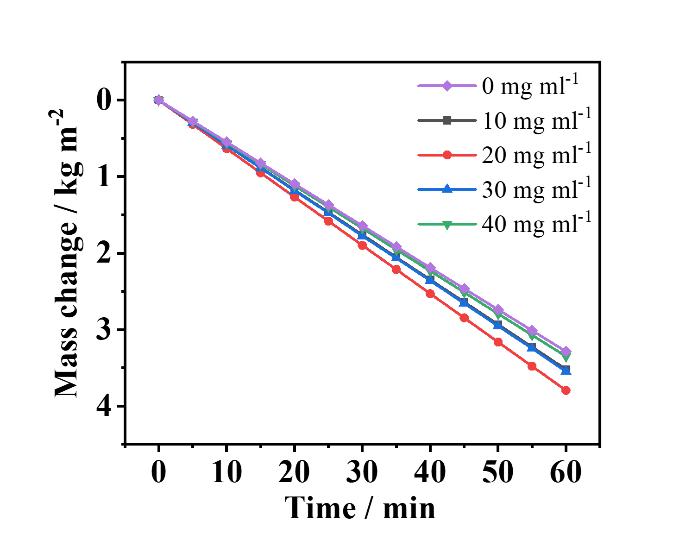


**Figure S10.** The evaporation performance of H-GCM@MS with different CDs contents.

**
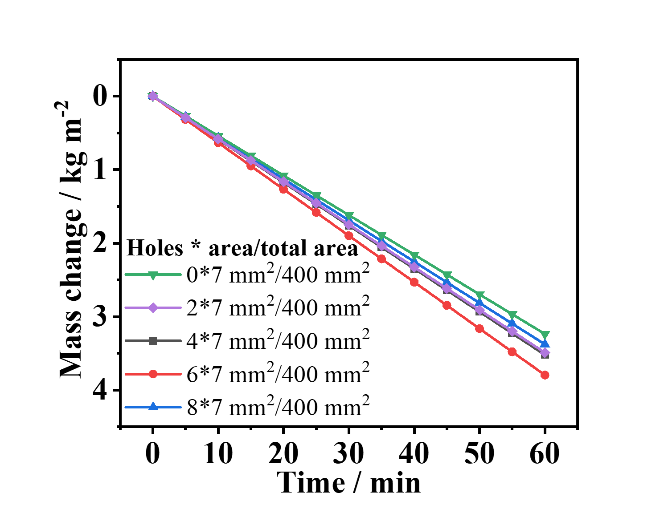
**

**Figure S11.** The evaporation performance of the H-GCM@MS with different number and area of the drilled channels.


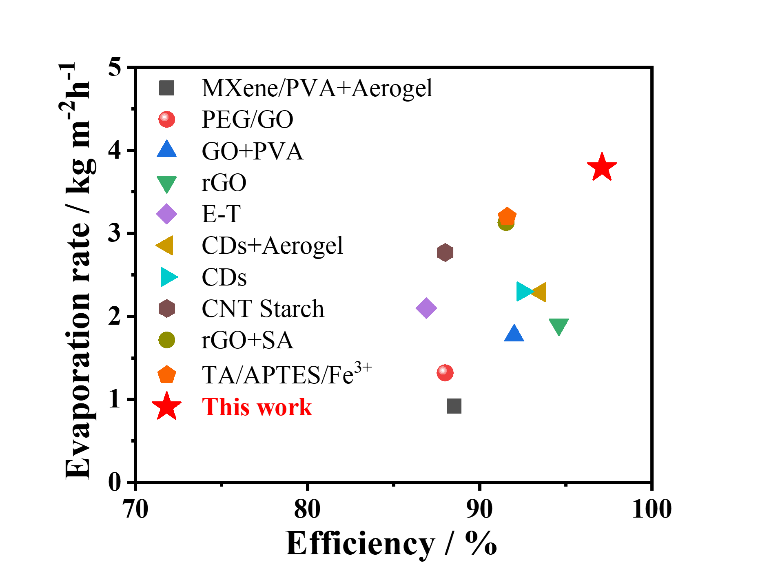


**Figure S12.** Comparisons of evaporation performances of H-GCM@MS with various evaporators reported in literature


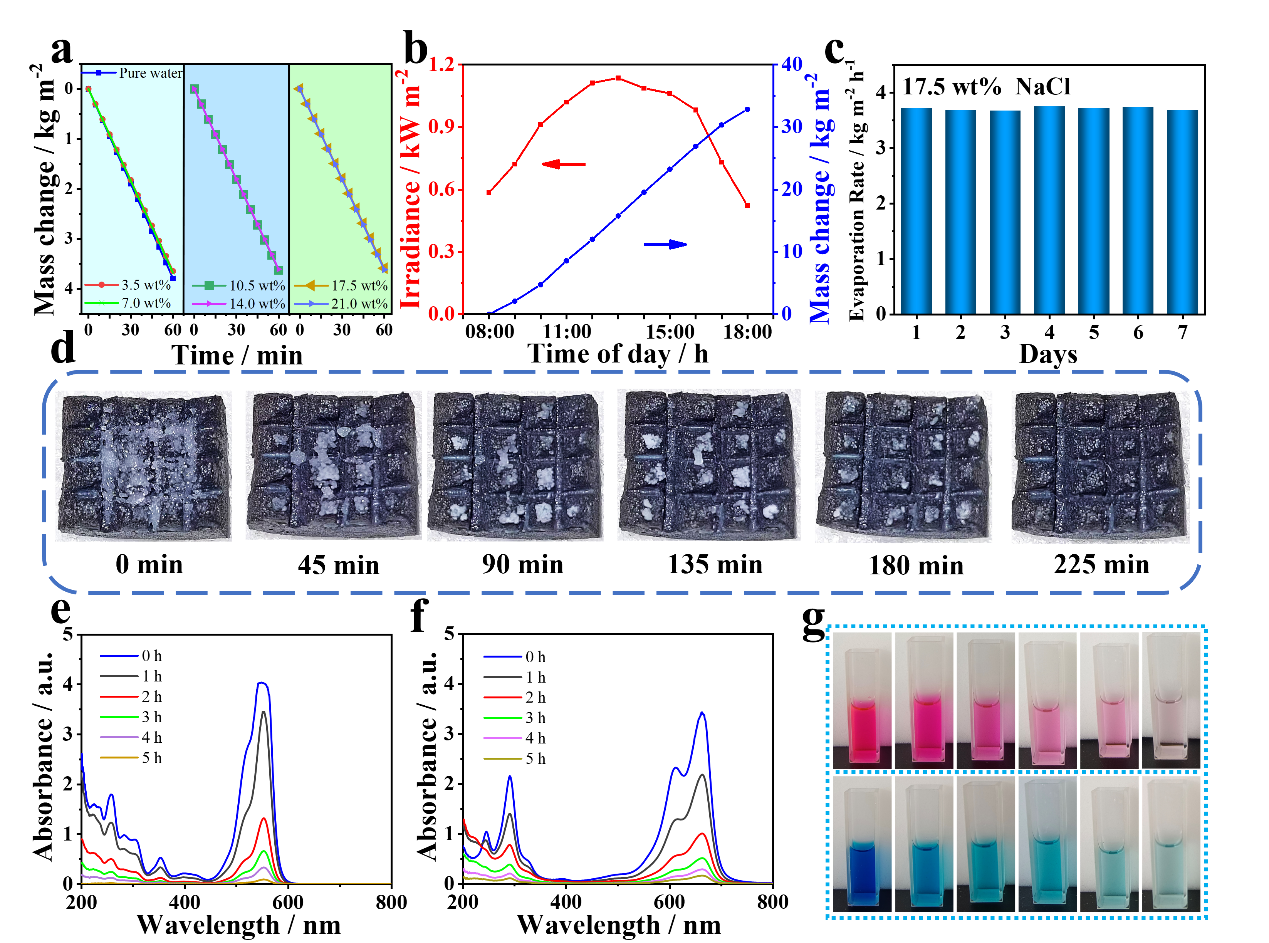


**Figure S13.** Salt-rejection performance and durability of H-GCM@MS. (a) Water evaporation performance of H-GCM@MS in different concentrations of brine; (b) The amount of purified water over time under natural sunlight and the intensity of solar radiation; (c) The stability and durability of H-GCM@MS in solar evaporation of 17.5% salt solution; (d) The evolution of the salt crystals placed on the surface of H-GCM@MS under natural evaporation; (e-g) UV-Vis absorption spectra of RhB and MB solution in the presence of H-GCM@MS and the corresponding photographs with different irradiation time.

Salt-resistant performance of the evaporator was examined in brine with salinity range of 0 to 21.0%. It was found that the impact of brine salinity on evaporation rates was insignificant (Figure S13a). To assess the salt-resistant performance of the evaporator under natural weather conditions, outdoor experiments were onducted, started at 8:00 (local time) and ended at 18:00 (local time Sep. 22, 2024). The evaporated water increased almost linearly with time (Figure S13b and S14). No salt formation on the surface of evaporator was observed. Moreover, the evaporation rate of brine with 17.5% salinity was maintained stable for up to 7 days (Figure S13c). The salt crystals placed on the evaporation surface were gradually dissolved, confirming the excellent salt-resistance performance of the evaporator (Figure S13d). In addition, the evaporator is also endowed with superior photocatalytic oxidation performance. When the evaporator is soaked in the solution containing organic contaminants such as methyl blue (MB) and rhodamine B (RhB), these contaminants can be rapidly decomposed (Figure S13e and f) and their colors were faded (Figure S13g) under solar irradiation. As thus, solar water evaporation and pollutant degradation can proceed simultaneously over the evaporator.

**
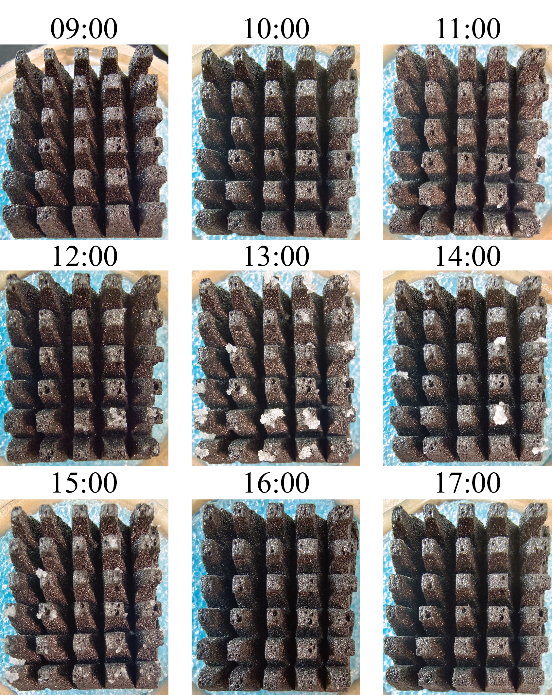
**

**Figure S14.** Photographs for outdoor salt resistance test of H-GCM@MS with 17.5 wt% salt solution. The test conditions is provided in Figure S13b

**
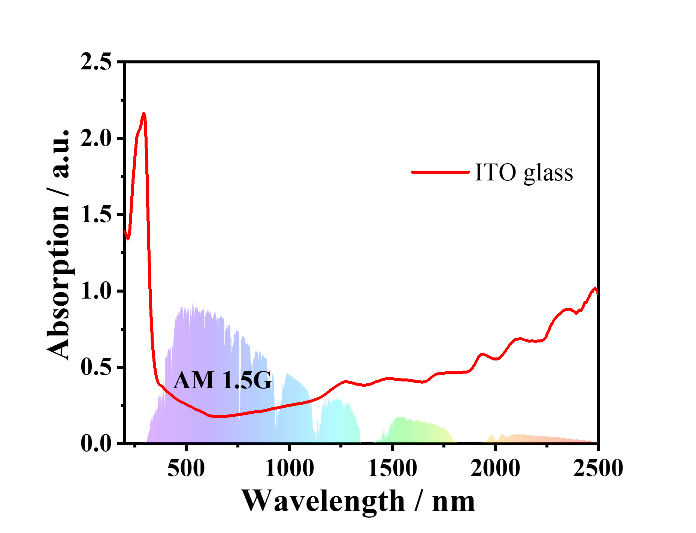
**

**Figure S15.** UV–vis–NIR absorption spectra of ITO glass

**
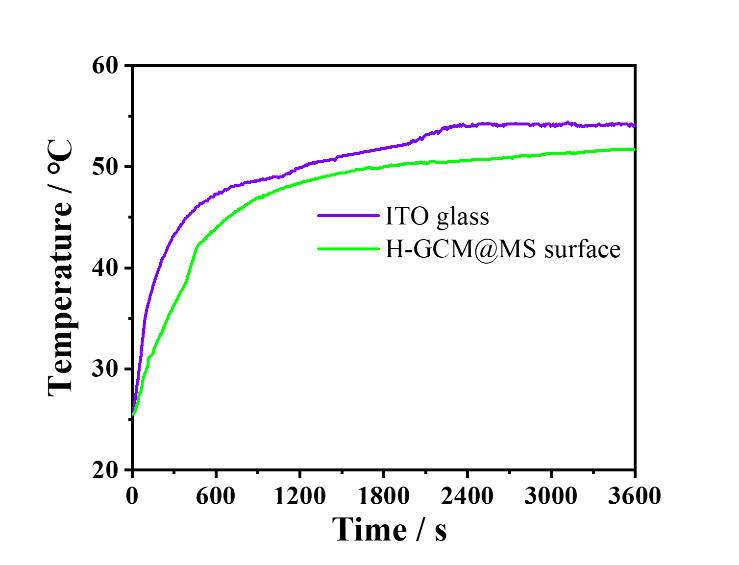
**

**Figure S16.** The surface temperatures of the evaporator and ITO glass

**
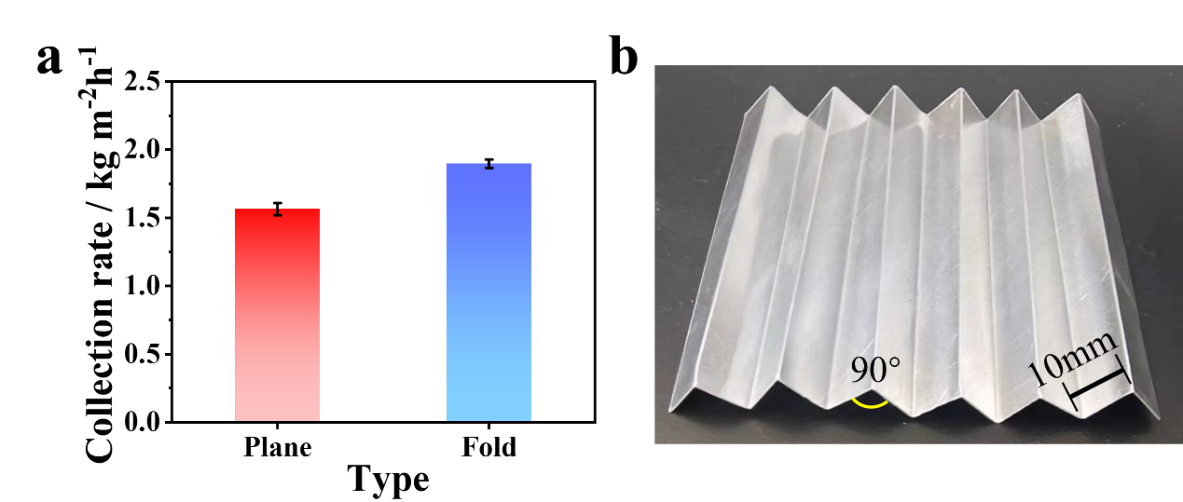
**

**Figure S17.** (a) The water collection rates of the Al sheet with different structures; (b) Photograph of the folded Al sheet

**
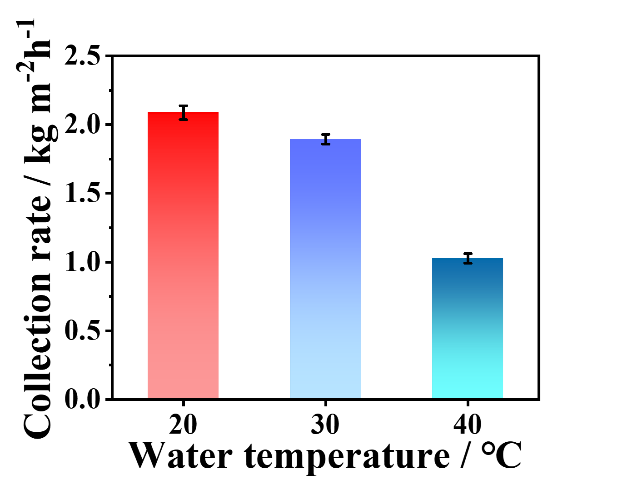
**

**Figure S18.** The influence of bulk water temperature on water collection rates

**
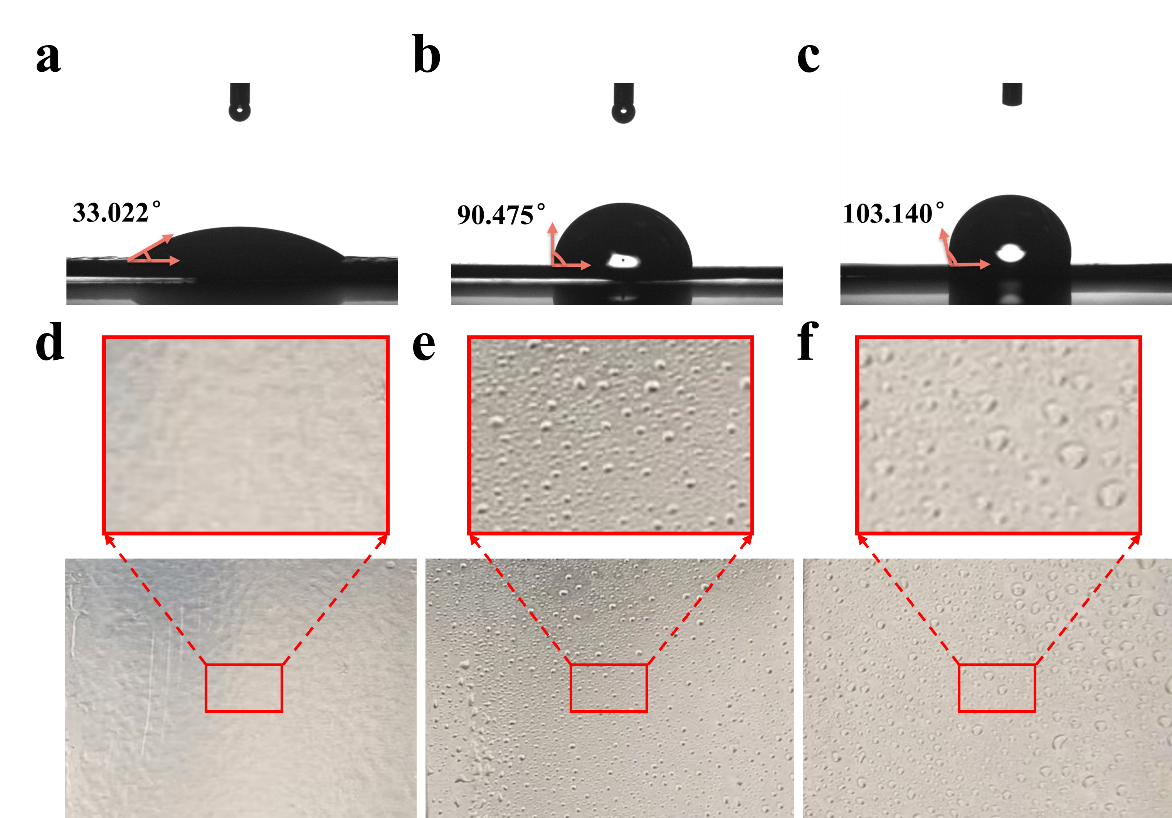
**

**Figure S19.** (a-c) Water contact angles on different substrates (a) Al surface coated with PEG, (b) pristine Al surface; (c) Al surface coated with PDMS; (d-f) The condensation phenomena of vapor on corresponding substrates

The condensation heat flux on the condenser can be expressed as:

$q_{c}$*=*$\frac{T_{\mathrm{vap}}-T_{\mathrm{amb}}}{R_{t}}$ (S10)

where *q*_c_ is the overall condensation heat flux of condensing chamber. *T*_vap_ and *T*_amb_ are the vapor temperature and ambient temperature, respectively. 𝑅_t_ is the equivalent thermal resistance of condensing chamber. Considering that the heat transfer is mainly fulfilled through the bottom condenser during vapor condensation process, the thermal resistance can be given as:

$R_{t}$*=*$\frac{{(T}_{\mathrm{in}}-T_{\mathrm{out}}) A_{c}}{Q}$ (S11)

where *T*_in_ and *T*_out_ are the inner surface temperature and outside surface temperature of the condenser, respectively, *A*_c_ is the contact area of condenser, *Q* is the transferred heat energy. So, the equation S11 can be expressed as,

$q_{c}$*=*$\frac{{(T}_{\mathrm{vap}}-T_{\mathrm{amb}}) Q}{{(T}_{\mathrm{in}}-T_{\mathrm{out}}) A_{c}}$ (S12)

It can be deduced that the overall condensation heat flux in condensing chamber depends on the *A*_c_. When water contact angle for the surface of condenser is larger, the *A*_c_ becomes smaller. This suggests that more condensation heat is transferred to the bulk water after introducing hydrophobic coatings. This phenomenon is also verified through the results of condensation rates at different bulk water temperatures (Figure S18).

**
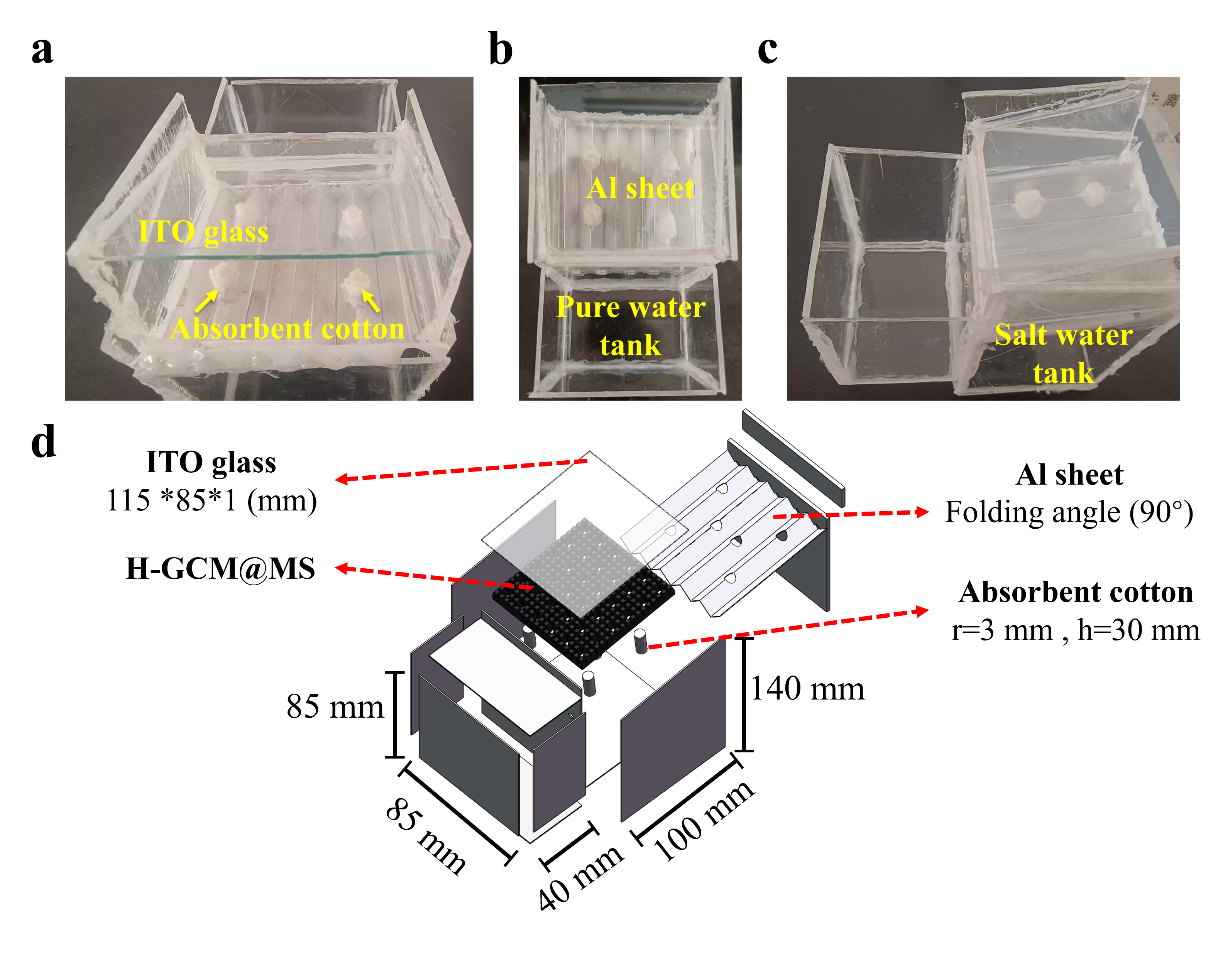
**

**Figure S20.** Schematic illustration of the parameters and structure of the solar evaporation-condensation device

**
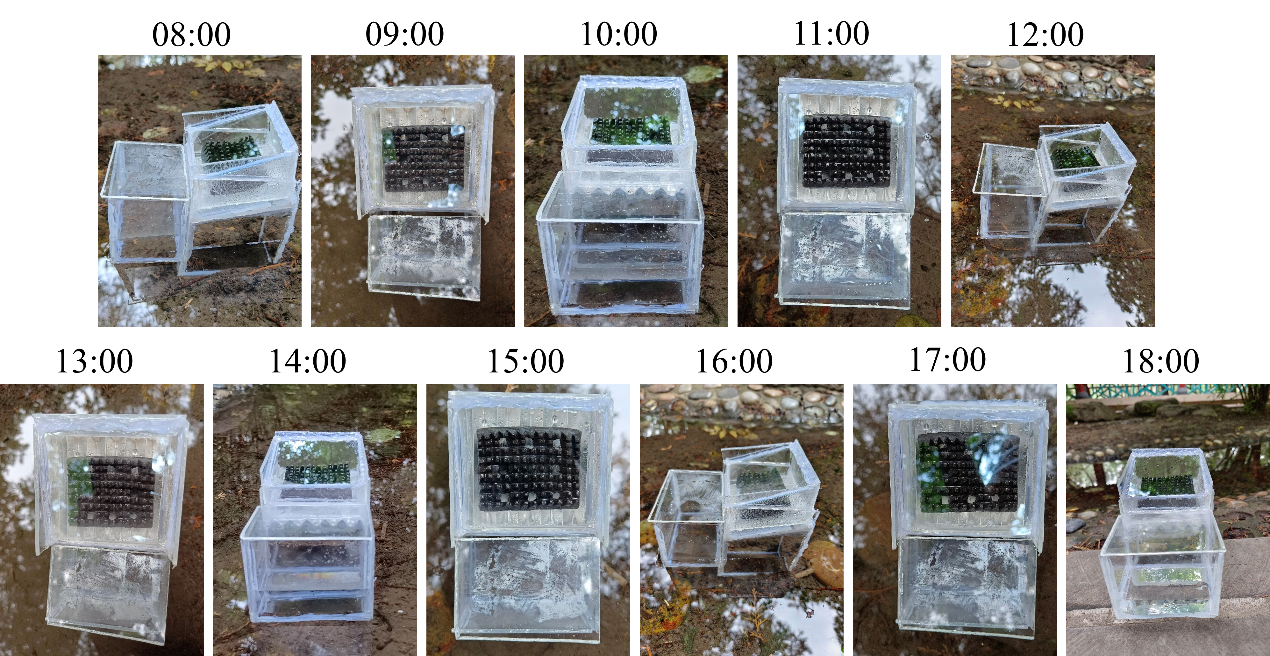
**

**Figure S21.** Outdoor water collect test of the device.

**
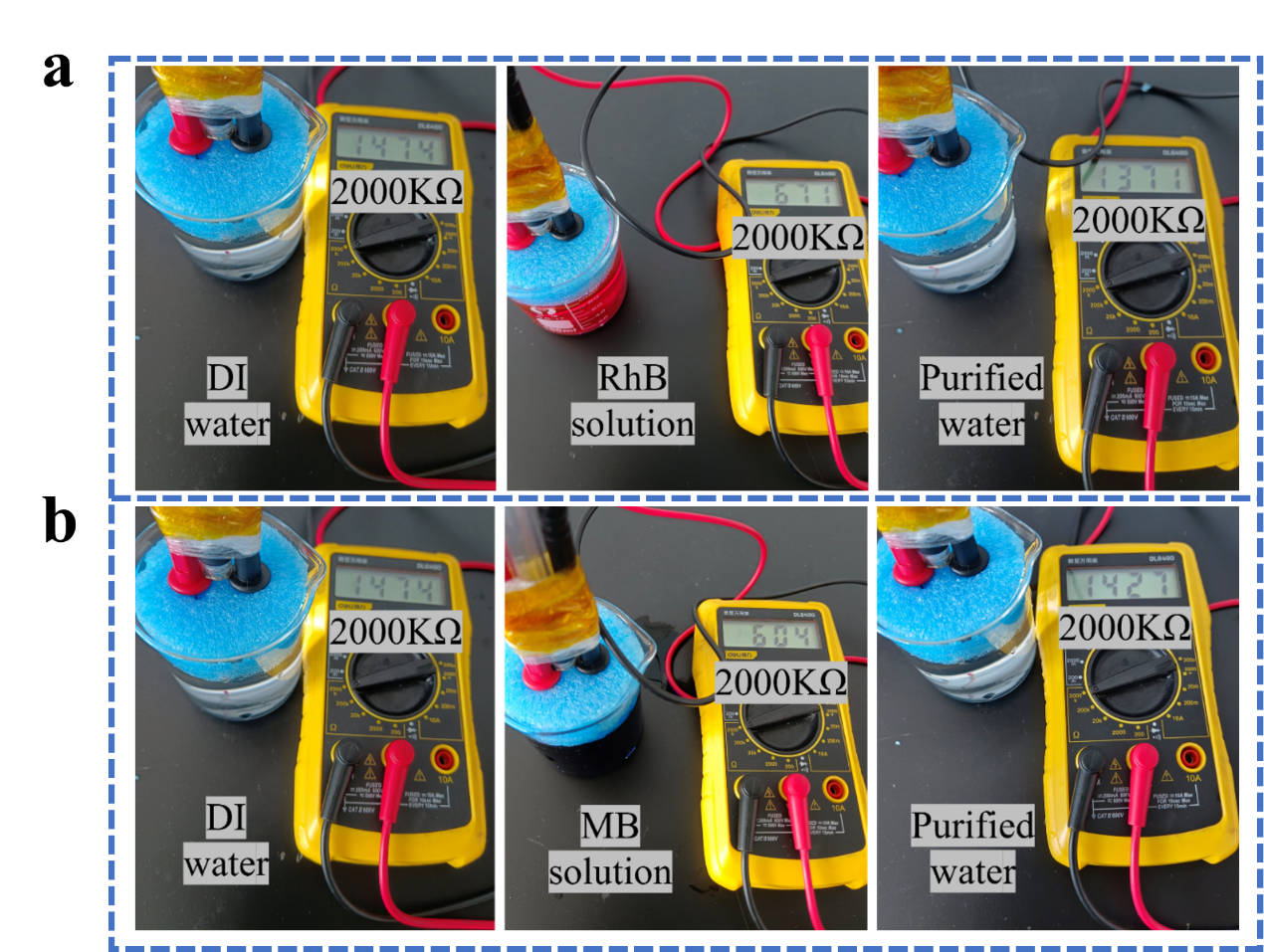
**

**Figure S22.** Comparison of resistance values before and after solar evaporation of contaminated water with RhB (a) and MB (b)

**Table S1.** Evaporation rates and photothermal conversion efficiencies of different evaporators

| **Evaporator type** | **Evaporation rate**  **(kg m^-2^ h^-1^)** | **Efficiency**  **(%)** |
| --- | --- | --- |
| Pyramid-like H-CDs@MS | 1.63 | 93.17 |
| Pyramid-like H-MXene@MS | 2.64 | 95.72 |
| Pyramid-like H-rGO@MS | 2.74 | 95.75 |
| Pyramid-like H-(MXene+CDs)@MS | 2.78 | 95.89 |
| Pyramid-like H-(rGO+CDs)@MS | 2.9 | 96.27 |
| Pyramid-like H-(rGO+MXene)@MS | 3.04 | 96.52 |
| Pyramid-like H-GCM@MS | 3.29 | 97.11 |

**Table S2.** Comparisons of evaporation performances of H-GCM@MS with various evaporators reported in literature

| **NO.** | **Evaporator carrier** | **Photothermal materials** | **Evaporation rate**  **(kg m^-2^ h^-1^)** | **Efficiency (%)** | **Ref.** |
| --- | --- | --- | --- | --- | --- |
| 1 | Aerogel | MXene/PVA | 0.92 | 88.52 | ^[S6]^ |
| 2 | Sponge | PEG/GO | 1.32 | 88 | ^[S7]^ |
| 3 | PVA | GO | 1.77 | 92 | ^[S8]^ |
| 4 | Sponge | rGO | 1.9 | 94.6 | ^[S9]^ |
| 5 | Sponge | E-T | 2.10 | 86.9 | ^[S10]^ |
| 6 | Aerogel | CDs | 2.29 | 93.5 | ^[S11]^ |
| 7 | Balsa wood | CDs | 2.3 | 92.5 | ^[S12]^ |
| 8 | Strach | CNT | 2.77 | 88 | ^[S13]^ |
| 9 | Sponge | rGO+SA | 3.13 | 91.54 | ^[S14]^ |
| 10 | Sponge | TA/APTES/Fe^3+^ | 3.2 | 91.6 | ^[S15]^ |
| **This work** | **Sponge+PVA** | **rGO+CDs+MXene** | **3.79** | **97.11** |  |

**Table S3.** Comparisons of collection performances of H-GCM@MS with various evaporators reported in literature

| NO. | Evaporation rate  (kg m^-2^ h^-1^) | Condensation  Technique | Collection rate  (kg m^-2^ h^-1^) | Efficiency (%) | Ref. |
| --- | --- | --- | --- | --- | --- |
| R1 | 2.63 | Single slope normal glass | 1.72 | 65.4 | ^[S16]^ |
| R2 | 1.12 | Single slope normal glass | 0.8 | 71.4 | ^[S17]^ |
| R3 | 2.19 | Single slope normal glass | 0.536 | 24.5 | ^[S18]^ |
| R4 | 3.2 | Single slope normal glass | 1.75 | 54.7 | ^[S15]^ |
| R5 | 1.83 | Single slope normal glass | 1.4 | 76.5 | ^[S6]^ |
| R6 | 1.69 | Double slope normal glass | 0.886 | 52.4 | ^[S19]^ |
| R7 | 2.42 | Semispherical normal glass | 1.46 | 60.2 | ^[S20]^ |
| R8 | 3.13 | Semispherical normal glass | 1.58 | 50.5 | ^[S14]^ |
| R9 | 3.53 | Conical normal glass | 1.27 | 36 | ^[S21]^ |
| R10 | 1.44 | Double slope PMMA | 0.64 | 45 | ^[S22]^ |
| R11 | 2.38 | Conical PMMA | 0.365 | 15.3 | ^[S23]^ |
| R12 |  | Single-stage invert-structured | 1.063 | 70 | ^[S24]^ |
| **This work** | **3.79** | **New inverted device** | **2.31** | **70** |  |

**References**

[S1] J. Su, Q. Chang, C. Xue, J. Yang, S. Hu, Electrochemical oxidation reconstructs graphene oxides on sponge for unprecedentedly high solar water evaporation, *Carbon* **2022**, *194*, 267-273.

[S2] X. Meng, Q. Chang, C. Xue, J. Yang, S. Hu, Full-colour carbon dots: From energy-efficient synthesis to concentration-dependent photoluminescence properties, *Chem. Commun.* **2017**, *53*, 3074-3077.

[S3] X. Fan, Y. Yang, X. Shi, Y. Liu, H. Li, J. Liang, Y. Chen, A mxene‐based hierarchical design enabling highly efficient and stable solar‐water desalination with good salt resistance, *Adv. Funct. Mater.* **2020**, *30*, 2007110.

[S4] L. Zhang, X. Li, Y. Zhong, A. Leroy, Z. Xu, L. Zhao, E. N. Wang, Highly efficient and salt rejecting solar evaporation via a wick-free confined water layer, *Nat. Commun.* **2022**, *13*, 849.

[S5] L. Li, C. Xue, Q. Chang, X. Ren, N. Li, J. Yang, S. Hu, H. Xu, Polyelectrolyte hydrogel‐functionalized photothermal sponge enables simultaneously continuous solar desalination and electricity generation without salt accumulation, *Adv. Mater.* **2024**, *36*, 2401171.

[S6] H. Zhang, X. Shen, E. Kim, M. Wang, J. H. Lee, H. Chen, G. Zhang, J. K. Kim, Integrated water and thermal managements in bioinspired hierarchical mxene aerogels for highly efficient solar‐powered water evaporation, *Adv. Funct. Mater.* **2022**, *32*, 2111794.

[S7] C. Liu, Y. Peng, X. Zhao, Continuous solar desalination based on restricted salt crystallization zone, *Desalination* **2021**, *501*, 114911.

[S8] W. Lei, S. Khan, L. Chen, N. Suzuki, C. Terashima, K. Liu, A. Fujishima, M. Liu, Hierarchical structures hydrogel evaporator and superhydrophilic water collect device for efficient solar steam evaporation, *Nano Res.* **2020**, *14*, 1135-1140.

[S9] J. Su, Q. Chang, C. Xue, J. Yang, S. Hu, Sponge‐supported reduced graphene oxides enable synergetic photothermal and electrothermal conversion for water purification coupling hydrogen peroxide production, *Sol. RRL* **2022**, *6*, 2200767.

[S10] H.-C. Li, H.-N. Li, L.-Y. Zou, Q. Li, P.-F. Chen, X.-N. Quan, K. Deng, C.-Q. Sheng, J. Ji, Q. Fan, Z.-K. Xu, J.-H. Wan, Vertically π-extended strong acceptor unit boosting near-infrared photothermal conversion of conjugated polymers toward highly efficient solar-driven water evaporation, *J. Mater. Chem. A* **2023**, *11*, 2933-2946.

[S11] X. Xu, Q. Chang, C. Xue, N. Li, H. Wang, J. Yang, S. Hu, A carbonized carbon dot-modified starch aerogel for efficient solar-powered water evaporation, *J. Mater. Chem. A* **2022**, *10*, 11712-11720.

[S12] Q. Hou, C. Xue, N. Li, H. Wang, Q. Chang, H. Liu, J. Yang, S. Hu, Self-assembly carbon dots for powerful solar water evaporation, *Carbon* **2019**, *149*, 556-563.

[S13] Y. Xu, B. Lv, Y. Yang, X. Fan, Y. Yu, C. Song, Y. Liu, Facile fabrication of low-cost starch-based biohydrogel evaporator for efficient solar steam generation, *Desalination* **2021**, *517*, 115260.

[S14] J. Xu, G. Wang, L. Zhu, G. Jiang, Y. Lei, Z. Zeng, L. Xue, Superwetting reduced graphene oxide/alginate hydrogel sponge with low evaporation enthalpy for highly efficient solar-driven water purification, *Chem. Eng. J.* **2023**, *455*, 140704.

[S15] Z. Wang, X. Wu, F. He, S. Peng, Y. Li, Confinement capillarity of thin coating for boosting solar‐driven water evaporation, *Adv. Funct. Mater.* **2021**, *31*, 2011114.

[S16] L. Wu, Z. Dong, Z. Cai, T. Ganapathy, N. X. Fang, C. Li, C. Yu, Y. Zhang, Y. Song, Highly efficient three-dimensional solar evaporator for high salinity desalination by localized crystallization, *Nat. Commun.* **2020**, *11*, 521.

[S17] D. Qi, Y. Liu, Y. Liu, Z. Liu, Y. Luo, H. Xu, X. Zhou, J. Zhang, H. Yang, W. Wang, X. Chen, Polymeric membranes with selective solution-diffusion for intercepting volatile organic compounds during solar-driven water remediation, *Adv Mater* **2020**, *32*, 2004401.

[S18] W. Ruan, H. Zhang, J. Fu, Z. Li, J. Huang, Z. Liu, S. Zeng, Z. Chen, X. Li, Z. Yu, X. Liang, J. Ma, Dissolution manufacturing strategy for designing efficient and low cost polymeric solar water evaporator, *Adv. Funct. Mater.* **2023**, *34*, 2312314.

[S19] H. Shan, Z. Ye, J. Yu, R. Wang, Z. Xu, Improving solar water harvesting via airflow restructuring using 3d vapor generator, *Device* **2023**, *1*, 100065.

[S20] H. Xie, Y. Du, W. Zhou, W. Xu, C. Zhang, R. Niu, T. Wu, J. Qu, Efficient fabrication of micro/nanostructured polyethylene/carbon nanotubes foam with robust superhydrophobicity, excellent photothermality, and sufficient adaptability for all‐weather freshwater harvesting, *Small* **2023**, *19*, 2300915.

[S21] X. Liu, F. Chen, Y. Li, H. Jiang, D. D. Mishra, F. Yu, Z. Chen, C. Hu, Y. Chen, L. Qu, W. Zheng, 3d hydrogel evaporator with vertical radiant vessels breaking the trade‐off between thermal localization and salt resistance for solar desalination of high‐salinity, *Adv. Mater.* **2022**, *34*, 2203137.

[S22] K. Yang, T. Pan, S. Dang, Q. Gan, Y. Han, Three-dimensional open architecture enabling salt-rejection solar evaporators with boosted water production efficiency, *Nat. Commun.* **2022**, *13*, 6653.

[S23] N. Liu, L. Hao, B. Zhang, R. Niu, J. Gong, T. Tang, Rational design of high‐performance bilayer solar evaporator by using waste polyester‐derived porous carbon‐coated wood, *Energy Environ. Mater.* **2021**, *5*, 617-626.

[S24] F. Wang, N. Xu, W. Zhao, L. Zhou, P. Zhu, X. Wang, B. Zhu, J. Zhu, A high-performing single-stage invert-structured solar water purifier through enhanced absorption and condensation, *Joule* **2021**, *5*, 1602-1612.
